# The intra-host evolutionary landscape and pathoadaptation of persistent *Staphylococcus aureus* in chronic rhinosinusitis

**DOI:** 10.1099/mgen.0.001128

**Published:** 2023-11-27

**Authors:** Ghais Houtak, George Bouras, Roshan Nepal, Gohar Shaghayegh, Clare Cooksley, Alkis James Psaltis, Peter-John Wormald, Sarah Vreugde

**Affiliations:** ^1^​ Adelaide Medical School, Faculty of Health and Medical Sciences, The University of Adelaide, Adelaide, Australia; ^2^​ The Department of Surgery - Otolaryngology Head and Neck Surgery, University of Adelaide and the Basil Hetzel Institute for Translational Health Research, Central Adelaide Local Health Network, Adelaide, Australia

**Keywords:** *S. aureus*, Chronic Rhinosinusitis, Biofilm, Evolution, Plasmids

## Abstract

Chronic rhinosinusitis (CRS) is a common chronic sinonasal mucosal inflammation associated with *Staphylococcus aureus* biofilm and relapsing infections. This study aimed to determine rates of *S. aureus* persistence and pathoadaptation in CRS patients by investigating the genomic relatedness and antibiotic resistance/tolerance in longitudinally collected *S. aureus* clinical isolates. A total of 68 *S *. *aureus* paired isolates (34 pairs) were sourced from 34 CRS patients at least 6 months apart. Isolates were grown into 48 h biofilms and tested for tolerance to antibiotics. A hybrid sequencing strategy was used to obtain high-quality reference-grade assemblies of all isolates. Single nucleotide variants (SNV) divergence in the core genome and sequence type clustering were used to analyse the relatedness of the isolate pairs. Single nucleotide and structural genome variations, plasmid similarity, and plasmid copy numbers between pairs were examined. Our analysis revealed that 41 % (14/34 pairs) of *S. aureus* isolates were persistent, while 59 % (20/34 pairs) were non-persistent. Persistent isolates showed episode-specific mutational changes over time with a bias towards events in genes involved in adhesion to the host and mobile genetic elements such as plasmids, prophages, and insertion sequences. Furthermore, a significant increase in the copy number of conserved plasmids of persistent strains was observed. This was accompanied by a significant increase in biofilm tolerance against all tested antibiotics, which was linked to a significant increase in biofilm biomass over time, indicating a potential biofilm pathoadaptive process in persistent isolates. In conclusion, our study provides important insights into the mutational changes during *S. aureus* persistence in CRS patients highlighting potential pathoadaptive mechanisms in *S. aureus* persistent isolates culminating in increased biofilm biomass.

## Impact Statement

Chronic rhinosinusitis (CRS) is characterised by chronic inflammation of the sinuses and nasal mucosal lining, causing symptoms such as nasal congestion, diminished sense of smell, facial pain, and breathing difficulties. Around 10 % of people worldwide suffer from CRS. The sinus microbiome affects the pathophysiology of CRS. *Staphylococcus aureus* is one of the most abundant species found in the sinuses and is associated with exacerbations of the condition. *S. aureus* biofilms are thought to modulate CRS, but little is known about the effect of *S. aureus* persistence in the sinonasal niche on biofilm formation and pathoadaptation. In this study, we evaluated the intra-host evolution of longitudinal *S. aureus* clinical isolates collected from the nasal cavities of subjects suffering from CRS. We used hybrid long and short read sequencing to assemble near reference-level genomes and linked this to *S. aureus* phenotypic changes in antimicrobial resistance and biofilm production *in vitro*. We show that persistent isolates were associated with an increase in biofilm tolerance against antibiotics and an increase in biofilm biomass over time. These isolates also had episode-specific mutational changes, often in host-adhesion genes. Additionally, we show that persistent isolates commonly undergo structural variation including host-adhesion gene recombination and in mobile genetic elements such as plasmids and prophages. We also found that the number of small nucleotide variants (SNVs) was not associated with the number of larger structural variations in persistent isolates. Furthermore, we demonstrate that plasmid copy number increases in the persistent strains, suggesting that traditional short-read based SNV approaches capture only a limited measure of genomic evolution and relatedness for within-host evolutionary studies. Overall, our study shows the value of hybrid bacterial genome sequencing in analysing within-host genomic evolution. Our approach, with its associated bioinformatics code included and documented, is useful not only for CRS, but is also widely applicable for any longitudinal bacterial genomics analysis.

## Data Summary

The assembled chromosomes and plasmids, raw short and raw long read FASTQ files are accessible on the Sequence Read Archive (SRA) under the project code: PRJNA914892. The complete list of biosample accession numbers for each sample can be found in Table S1, available in the online version of this article. All code used to generate all analyses & figures used in this manuscript can be found at https://github.com/gbouras13/CRS_Saureus_Evolutionary_Landscape.

## Introduction

Chronic rhinosinusitis (CRS) is characterised by ongoing inflammation of the paranasal sinuses and nasal mucosal lining, which causes symptoms such as nasal congestion, diminished sense of smell, facial pain, and breathing difficulties [1]. Around 10 % of people worldwide suffer from CRS, making it a common condition [2].

CRS is clinically subdivided based on its phenotype into two subcategories, CRS with nasal polyps (CRSwNP) and CRS without nasal polyps (CRSsNP) [3]. Although the pathogenesis of CRS remains unknown, it is known to be a heterogeneous multi-factorial chronic inflammatory disease that frequently co-occurs with conditions such as ciliary dysfunction, aspirin-exacerbated respiratory disease (AERD), and asthma [1].

It is thought that microbes impact the pathophysiology of CRS. One of the bacteria most abundantly found in the sinuses of CRS patients is *Staphylococcus aureus *, which is frequently cultured from the nose during the acute exacerbations of the condition [4, 5].

Several mechanisms of involvement of *S. aureus* in the pathophysiology of CRS have been proposed, including *S. aureus* biofilms as a modulator of chronic mucosal inflammation and relapsing infections [5, 6]. Moreover, *S. aureus* mucosal biofilms are associated with poor post-surgical outcomes in patients undergoing functional endoscopic sinus surgery to treat their CRS-associated sinus symptoms [7, 8].

Despite the lack of high-level evidence for the effectiveness of antibiotics in treating CRS and its exacerbations, they are commonly prescribed to CRS patients [1, 9]. Moreover, antibiotics are often ineffective at eliminating the biofilm nidus resulting in a relapsing course of infectious exacerbations [10].

Previously we showed with pulsed-field gel electrophoresis that it is common for subjects suffering from CRS to be persistently colonised over several months by *S. aureus* even when undergoing multiple courses of antibiotics [11]. This suggests that the bacteria can persist in the sinuses despite antibiotic treatment. However, what is less clear is the pathogenic adaptation and phenotypic changes that occur during chronic infection of difficult-to-treat CRS patients. While *S. aureus* persistence in the nasal cavity is generally considered a commensal state, its significance changes in the context of CRS, where it is associated with poorer post-surgical outcomes. Moreover, studies suggest that *S. aureus* plays a crucial role in modulating type two polarized airway inflammation, which is frequently observed in CRS. This involvement is attributed to various mechanisms, such as the release of IL-33 from respiratory epithelium, activation of innate lymphoid cells, formation of IgE, and attraction of eosinophils [12]. Pathogenic adaptation may occur through the acquisition of host immune evasion genes or the ability to form biofilms, further exacerbating its pathogenic potential.

This study aimed to evaluate the intra-host relatedness of longitudinal *S. aureus* clinical isolates (CI) collected from the nasal cavities of subjects suffering from CRS and characterise the adaption that enables persistence in the host using hybrid long and short read assembled reference-level genomes. Furthermore, intra-, and inter-host variability in *S. aureus* phenotype regarding antimicrobial resistance and biofilm tolerance to antibiotics was evaluated to identify phenotypic pathoadaptation of persistent strains.

## Methods

### Clinical isolate retrieval

*S. aureus* clinical isolates were retrieved from a bacterial biobank comprised of samples stored in 25 % glycerol stock at −80 °C, obtained from swabs taken from the sinonasal cavity of subjects. The swabs were collected from ear-nose-throat inpatient clinic follow-ups and during sinonasal surgery. The swabs were processed (purified and re-streaked) at a commercial microbiology laboratory (SA Pathology, Adelaide, Australia) or in-house. The pathology reports did not indicate the co-colonization of *S. aureus *. All swabs processed in-house were screened on co-colonisation based on morphology and colour of colonies. All isolated strains were stored in 25 % glycerol stock at −80 °C.

To be included in this study, longitudinal clinical isolate pairs had to be isolated from swabs obtained from patients who fulfilled the EPOS 2020 criteria for difficult-to-treat CRS [1]. The diagnostic criteria and retrieval of asthma, aspirin sensitivity and CRS subtype are elaborated in supplementary text ST1.

Only clinical isolate pairs with a time difference of over 5 months between collections were included in the study. When a subject had more than two clinical isolates available at different timepoints, the isolated pair with the largest time difference was selected. We termed the first recovered isolate group T0, whereas the isolates recovered at later timepoints were termed T1. For all experiments, the clinical isolates were grown overnight on nutrient agar plates (Thermo Fisher Scientific, CM0003, Waltham, USA) from glycerol stock at 37 °C unless otherwise specified.

### Antibiotic exposure

The antibiotic exposure of subjects was assessed based on the antibiotic scripts in their medical records. All antibiotic treatments prescribed to the subjects between their first and second sample collection were extracted. The total antibiotic exposure was calculated as the cumulative number of days prescribed for the treatments [13].

### Genomic DNA extraction and sequencing

For all clinical isolates, hybrid long and short sequencing was performed. The genomic DNA of the *S. aureus* clinical isolates was extracted using the DNeasy Blood and Tissue Kit (Qiagen, 69 504, Hilden, Germany) following the manufacturer’s guidelines. The extracted DNA was sequenced using the Oxford nanopore technology (ONT) on the MinION Mk1C (Oxford Nanopore Technologies, Oxford, UK) for long-read sequencing. The Rapid Barcoding Kit (Oxford Nanopore Technology, SQK-RBK 110.96) was used to sequence the long-read *S. aureus* whole genome on R9.4.1 MinION flowcells (Oxford Nanopore Technology), using 50 ng of the isolated DNA. Base-calling was conducted with Guppy v 6.2.11 in super accuracy mode, using the 'dna_r9.4.1_450bps_sup.cfg' configuration (Oxford Nanopore Technology). The short-read sequencing was done at a commercial sequencing facility (SA Pathology, Adelaide, SA, Australia) as previously described by Shaghayegh *et al*. [14]. Short-read sequencing was conducted on the Illumina platform, using the Illumina NextSeq 550 (Illumina Inc, San Diego, USA) and NextSeq 500/550 Mid-Output kit v2.5 (Illumina Inc., FC-131–1024). To prepare for short-read sequencing, the genomic DNA was isolated using the NucleoSpin Microbial DNA kit (Machery-Nagel GmbH and Co.KG, 740 235.50, Duren, Germany). The sequencing libraries were prepared using a modified protocol for the Nextera XT DNA library preparation kit (Illumina Inc. FC-131–1024). The genomic DNA was fragmented, after which a low-cycle PCR reaction was used to amplify the Nextera XT indices to the DNA fragments. One hundred fifty bp reads were obtained by sequencing after manual purification and normalisation of the amplicon library.

### Bioinformatics

#### Chromosome assembly

We created complete chromosomal assemblies of *S. aureus* using a custom Snakemake pipeline [15] available as a Snaketool [16] powered command line program called hybracter that can be accessed via https://github.com/gbouras13/hybracter [17, 18]. Briefly, the long reads were reduced to 250 Mbp for each sample using Rasusa v0.7.0 [19]. Long reads were filtered using Filtlong v0.2.1 [20]. Adapters and barcodes were removed using Porechop v0.2.4 [21], short reads were filtered and trimmed with low-quality regions, and adapters were removed using fastp v0.18.4 [22]. Long-read-only assemblies were created using Flye v2.9.1 with the option ‘--nano-hq.’ [23]. Assemblies, including contigs with a length greater than 2.5 Mb, were kept and denoted as the putative chromosomal. The resulting chromosomes were first polished with long reads using Medaka v1.7.0 [24]. After the first round of polishing, the chromosomes were reoriented to start at the *dnaA* gene using dnaapler v0.0.1 [25]. Finally, chromosomes were polished for a second time with long-reads using Medaka v1.7.0, then with Polypolish v0.5.0 [26] and POLCA v4.1.0 [27]. Fig. S1 shows a workflow diagram of the assembly pipeline.

#### Plasmid assembly

Plassembler v 0.1.4 [28] was used to assemble bacterial plasmids from a combination of long and short sequencing reads. Firstly, the short reads are filtered using fastp. The long reads were filtered using nanoFilt v.2.7.0 [29] and then assembled using Flye v2.9.1. The largest contig was evaluated to see if the assembly contained more than one contig. If this contig was over 90 % of the length of the chromosome size (~2.5 MB), it was identified as the chromosome. All other contigs were deemed putative plasmid contigs. Both long and short reads were then mapped twice, first to the chromosome and then to the plasmid contigs. For the mapping, minimap2 v2.24 [30] was used for the long reads, while BWA-MEM v0.7.17 [31] was used for the short reads. Reads aligned to the plasmid contigs or not aligned to the chromosome were extracted, combined, and de-duplicated. To produce the final plasmid contigs, these reads were assembled using Unicycler v0.5.0 [32].

#### Annotation

Chromosome and plasmid assemblies were annotated with Bakta v1.5.0 [33]. The assemblies were typed according to multi-locus sequence typing (MLST) using the programme mlst v2.23 [34] and assigned to clonal complexes of PubMLST [35]. Variable-length-k-mer clusters (VLKCs) were used to query the assemblies with k-mer lengths ranging from 13 to 28 and a sketch size of 9984 using the pp-sketchlib tool with PopPUNK v2.6.0 [36]. The VLKCs were assigned to the pre-built PopPUNK Staphopia database of 103 clusters for phylogenetic analysis [37]. The phylogenetic tree was visualised using the ggtree R package [38]. Additionally, a maximum-likelihood phylogenetic tree was constructed using the core genome alignment from panaroo v1.3.2 specifying ‘--clean-mode strict -a core --aligner mafft --core_threshold 1 --remove-invalid-genes’ [39] with IQ-TREE v 2.2.0.3 specifying ‘-m GTR +R’ (i.e. GTR plus freerate) [40] as the model.

#### Chromosome analysis

The presence or absence of resistance and virulence genes in the genome of the clinical isolates was determined by screening contigs using ABRicate v1.0.1 [41] against the Comprehensive Antibiotic Resistance Database (CRAD) [42] and the Virulence Factor Database (VFDB) [43].

Genome-wide association analysis was done by first creating a pangenome of the 34 T0 isolates with panaroo v1.3.2 [39] and then testing the significance of each gene with Scoary v1.6.16 using default parameters [44]. All following paired *S. aureus* genomic analysis was conducted using a Snakemake pipeline. Firstly, small variants, such as single nucleotide variants (SNVs) and small insertions and deletions, were called using Snippy v 4.6.0 [45], with the raw FASTQ short reads from the Timepoint T1 isolates were compared against the corresponding GenBank file of the assembled Timepoint T0 isolate for each clinical isolates pair. All larger structural differences were called using two methods: Nucdiff v2.0.3 [46] and Sniffles v2.0.7 [47]. For Nucdiff, chromosome assembly of the T0 isolate was compared against the corresponding T1 isolate. For, Sniffles, all T1 isolate long reads were first aligned to the T0 isolate genome using minimap2 v 2.24 [30] specifying '-ax map-ont' parameters. The resulting BAM was used as input for Sniffles.

The large structural variant clinical isolates pairs of subject 420 and 4875 were manually annotated by mapping all timepoint T1 long reads to the T0 assembly using minimap2 v 2.24 specifying '-ax map-ont', followed by sorting the resulting BAM file using SAMtools v1.17 [48]. Structural deletions were visualised in R using the gggenomes, and the long-read pile-up was visualised using the Gviz packages [49, 50]. Fig. S2 shows a workflow diagram of the chromosomal analysis.

#### Plasmid analysis

For each putative plasmid contig derived from the output of Plassembler, Mobtyper v1.4.9 [51] was run to determine each plasmid’s predicted mobility and replicon marker. The minhash ('Mash') distance was calculated between each pair of plasmids using mash v2.3 [52]. A plasmid pangenome was created using panaroo v1.3.2. To determine shared plasmid genes using the ‘gene_presence_absence. Rtab’ output, the Jaccard index based on gene presence and absence, was calculated between each plasmid pair. Following the analysis by Hawkey *et al*., plasmids were empirically determined to be the same plasmid using thresholds of Mash similarity >0.98 and Jaccard index >0.7 [53]. Additionally, plasmids were determined to be beta-lactamase-carrying if they carried the *blaZ*, *blaI* and *blaR1* gene operon. All plasmid-copy numbers were obtained using Plassembler v0.1.4. Fig. S3 shows a workflow diagram of the plasmid analysis.

### MSCRAMM coding sequence proportion, codon usage bias, and dN/dS estimation

To calculate the proportion of coding sequences comprised of MSCRAMM genes, we considered 19 possible MSCRAMM genes identified in our Panaroo pangenomes (Table S2). We then calculated the average size of the 19 genes in base pairs (taking an average gene size output from Panaroo), summed them and divided it by our estimate for the total coding bases in *S. aureus *. Using an approximate coding density of 85 % and an approximate average chromosome length of 2.8 MB, this yielded a total MSCRAMM coding sequence proportion of around 2 % of all *S *. *aureus* coding nucleotides.

To provide insight into the evolutionary forces acting on protein-coding genes the ratio of non-synonymous (dN) to synonymous (dS) changes was used as a measure of selection strength and direction [54]. To calculate the dN/dS ratio, a baseline estimated rate of non-synonymous mutation of 4.6 times higher than that of synonymous mutation in *S. aureus* was taken from [55]. The observed non-synonymous to synonymous mutation ratio in the same-strain isolates was 104/44=2.36, thereby yielding an estimated dN/dS ratio of 2.36/4.6=0.51. To approximately assess codon usage differences between MSCRAMM and non-MSCRAMM genes, a custom Python script ‘ calc_ codon_ bias. py’ available in the CRS_Saureus_Evolutionary_Landscape GitHub repository was used. This script calculates the number of possible non-synonymous to synonymous nucleotide changes for each codon, then averages it across all codons for an input multiFASTA containing coding sequences.

### Relatedness of isolate pairs

A two-step approach was used to classify isolate pairs as either closely related ‘same strain’ or not closely related ‘different strain’. Firstly, the Sequence Types obtained from MLST and clusters generated by PopPUNK were compared between each isolate pair. If either of these metrics differed in the clinical isolate pair, they were considered to belong to different strains. The second step involved analysing the mutation rate based on the number of single nucleotide variants (SNVs) outputted by Snippy. Isolate pairs with fewer than 2.5×10^− 5^ mutations per nucleotide per year between the first and second timepoint were classified as the same strain. This cutoff value was determined based on the mutation rates reported in various studies for *S. aureus *, which ranges from 1.22×10^− 6^ to 3.30×10^− 6^ mutations per nucleotide per year [56–63]. It’s important to note that this range accounts for potential variations in SNVs between pairs, given that we do not have mutation rate confidence intervals available for the specific isolate pairs under investigation.

### Planktonic antibiotic susceptibility

Susceptibility testing followed Clinical and Laboratory Standards Institute (CLSI) guidelines (CLSI, 2020). Seven antibiotics were chosen for susceptibility testing according to their common use in medical practice. These were: amoxicillin in combination with clavulanic acid (augmentin), clarithromycin, clindamycin, doxycycline, erythromycin, gentamicin, and mupirocin (Sigma-Aldrich, St. Louis, USA). Minimum Inhibitory Concentrations (MICs) were obtained for the planktonic form of all isolates, using the microbroth dilution assay [64]. The antibiotics were tested at 0.06–32 mg l^−1^ dilution range. The assay was repeated at least twice per CI. The MIC50, MIC90 and antibiotic non-susceptibility proportions were calculated adopting the susceptibility breakpoints published by the CLSI.

### Biofilm antibiotic tolerance

The biofilm tolerance assay was based on a 96-well plate adapting the procedures used by Mah *et al*. [65]. Each isolate was exposed to the same antibiotics used for the planktonic antibiotic susceptibility testing. The concentrations ranged from 1.25 to 640 mg l^−1^. In brief, the clinical isolates were cultured on Mueller-Hinton agar (Sigma-Aldrich). Then, single colonies of *S. aureus* were suspended in 0.9 % saline to a turbidity reading of 0.5 McFarland Units (MFU). The 0.5 MFU bacterial suspension was diluted 100-fold in Mueller-Hinton broth to achieve a 5×10 ^5^ c.f.u. ml^−1^ before inoculation in a 96-well plate (200 µl). Plates underwent a 48 h incubation at 37 °C with sheer force on a rotating plate set at 70 r.p.m. (3D Gyratory Mixer, Ratek Instruments, Australia). Following the incubation, the supernatants were gently aspirated with a minimum agitation of the biofilms. These biofilms were then exposed to different antibiotics in serial diluted (200 µl) Mueller-Hinton broth for 24 h. After incubation with antibiotics, the supernatants were aspirated gently, and non-adherent planktonic bacteria were removed by gently washing with sterile phosphate-buffered saline (PBS). Subsequently, the biofilm tolerance was assayed using a resazurin viability method, alamarBlue Cell Viability Reagent (Thermo Fisher Scientific, DAL1025), as per the manufacturer’s instructions [66]. The assay was repeated twice per clinical isolate with two replicates.

### Biofilm biomass assay

To quantify the total biofilm biomass, the Crystal Violet (CV) staining assay was used [67]. Inoculated 96-well plates underwent a 48 h incubation at 37 °C on a rotating plate set at 70 r.p.m. to induce biofilm formation. Following the incubation, the planktonic cells were removed by gently aspirating the supernatants and washing the wells twice with PBS. Subsequently, 200 µl of 0.1 % CV (Sigma-Aldrich, C6158) solution was added for 15 min. After washing the wells three times with sterile water and air-drying, the fixed CV was solubilised by adding 200 µl 30 % acetic acid and shaking for 1 h at room temperature. The absorbance was obtained at 595 nm with a FLUOstar Omega microplate reader (BMG Labtech, Ortenberg, Germany). The assay was repeated twice per strain, with six technical replicates.

### Statistics

We used a generalised linear mixed model (GLMM) to analyse the antibiotic tolerance data. The threshold of significance was set at a *p*-value<0.05. All analysis was performed with R v4.2.0 [68].

## Results

### Clinical characteristics

Thirty-four *S. aureus* sequential pairs (68 clinical isolates from which 34 first timepoint [T0] and 34 s [T1] isolates) were included in this study, isolated from 34 subjects. The mean time between paired *S. aureus* clinical isolate collection was 415 days (range 184–1561) (Table 1). Most subjects were classified as CRSwNP (85%) and having asthma (56%). The clinical characteristics of the subjects are summarised in Table S3.

**Table 1.** Time between isolate pair collection

Host ID	T0 isolate	T1 isolate	Time between isolate pairs (days)
**276**	C100	C364	1318
**420**	C22	C320	1283
**539**	C235	C318	310
**1170**	C79	C148	243
**1415**	C265	C324	294
**1676**	C13	C76	245
**1992**	C80	C208	580
**2911**	C240	C295	184
**3344**	C52	C113	202
**3357**	C24	C195	772
**3997**	C9	C353	1561
**4009**	C121	C255	712
**4681**	C3	C56	189
**4747**	C32	C188	691
**4784**	C16	C70	201
**4875**	C67	C294	987
**4986**	C21	C273	1087
**5047**	C72	C351	1344
**5060**	C133	C179	247
**5142**	C45	C149	431
**5308**	C91	C209	553
**5328**	C206	C276	316
**5390**	C136	C197	357
**5448**	C155	C339	894
**5469**	C196	C342	687
**5485**	C183	C312	581
**5503**	C182	C233	301
**5519**	C224	C349	646
**5562**	C222	C333	567
**5631**	C245	C314	238
**5647**	C285	C355	399
**5728**	C241	C309	236
**5767**	C325	C363	231
**5911**	C311	C357	288

### *S. aureus* strains persist within the sinonasal cavities in 41% of cases

**Fig. 1.**
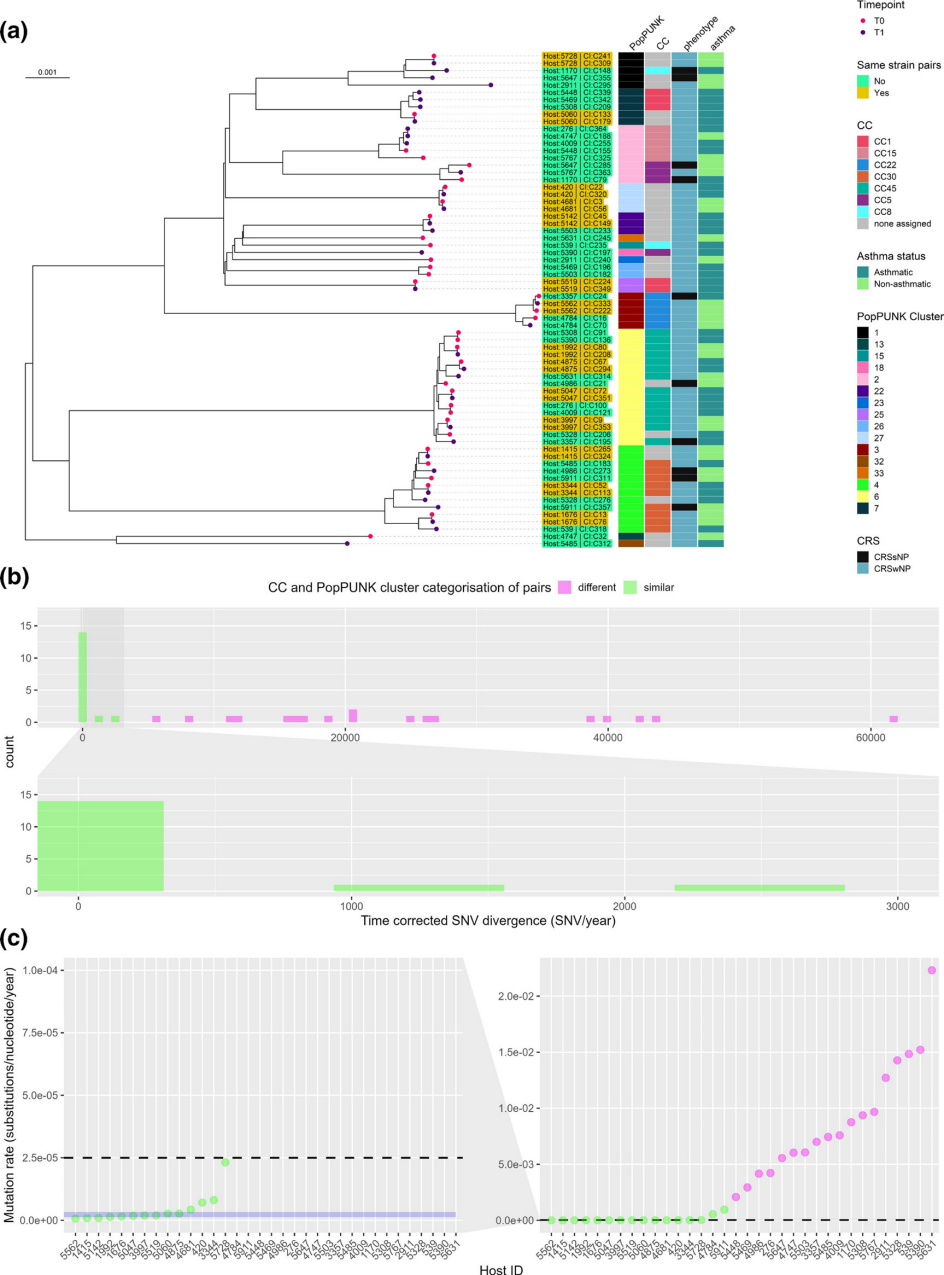
Genome-based classification of *Staphylococcus aureus* clinical isolates. (**a**) A PopPUNK variable-length-k-mer cluster (VLKC) midpoint rooted tree of 68 *S *. *aureus* genomes collected from 34 subjects with chronic rhinosinusitis (two samples per subject) based on PopPUNK analysis. The branch tip colours represent the collection timepoint (T0=first, T1=later timepoint). The PopPUNK VLKC, clonal complex (CC), CRS phenotype, and asthma status are indicated by colour on the right side. The branch labels show the corresponding host ID and the clinical isolate number colour coded with same or different strain pair classification. (**b**) A histogram depicting the distribution of time corrected pairwise single-nucleotide variant (SNV) divergence (total number of SNV/time between isolates in years) in the core genome for all clinical isolate pairs (*n*=34), with colours indicating CC and VLKC categorisation between pairs. (**c**) Mutation rates (substitutions per nucleotide per year) for all bacterial isolates pairs (*N*=34). The horizontal dashed line indicates the mutation rate threshold of 2.5×10^− 5^ mutations/nucleotide/year used to classify pairs as either ‘same strain’ or ‘different strain’. The blue area represents the mutation rates as reported for *S. aureus* in various other studies (1.22×10^− 6^ to 3.30×10^− 6^ mutations per nucleotide per year) [56–63].

The MLST analysis revealed a total of seven clonal complexes (CCs), including CC1 (*n*=5, 7.3 %), CC5 (*n*=4, 5.8 %), CC8 (*n*=2, 2.9 %), CC15 (*n*=5, 7.3 %), CC22 (*n*=5, 7.3 %), CC30 (*n*=9, 13.2 %), and CC45 (*n*=14, 20.5 %). A total of 24/68 clinical isolates (35.2 %) were not assigned to any CC (Fig. 1a). The analysis of the PopPUNK variable-length-k-mer clusters (VLKCs) identified a total of 16 clusters. Of the 34 isolate pairs, 18 (52.9 %) pairs belonged to different CCs or VLKCs, indicating that they were not closely related isolates and were classified as ‘different strain’ pairs.

The relatedness of the remaining 16 pairs was examined by assessing the mutation rate between the pairs of isolates. In Fig. 1b, the distribution of time-corrected SNVs (total number of SNVs/years between isolate pairs) between all clinical isolate pairs is displayed. Among the 16 pairs, the count of time-corrected SNVs varied from 1.9 to 2690.6. Notably, 14 out of the 16 pairs exhibited a mutation rate of 2.3×10^−5^ mutations per nucleotide per year or lower, while two out of the 16 pairs had a total number of SNVs between them exceeding the cutoff threshold of 2.5×10^− 5^ mutations per nucleotide per year. This threshold corresponds to the expected mutation rate between closely related ‘same strain’ pairs based on previously reported data [56–63, 69]. In Fig. 1c, the mutation rates of all isolate pairs are depicted and reflect the classification of pairs into different and same strain groupings. Two pairs, identified as having the same Clonal Complex (CC) and PopPUNK cluster with a total number of SNVs between pairs exceeding 2.5×10^− 5^ mutations per nucleotide per year (host 4784 with 1540 time-corrected SNVs and host 5911 with 2690 time-corrected SNVs), were classified as ‘different strain’ pairs. These two isolate pairs also exhibited a notable number of structural variations. Specifically, the clinical isolate pair from host 4784 displayed 100 structural variations between time points T0 and T1 as determined by Sniffles. Notably, a plasmid was introduced in the T1 isolate. Similarly, the clinical isolate pair originating from host 5911 showed 143 structural variations between them. Accordingly, 14/34 pairs (41%) were classified as in the ‘same strain’ group of persistent isolates, whilst 20/34 pairs (59 %) were classified as being part of the ‘different strain’ group, where the subject had been colonised or infected by a different strain over time (Table S4). These 14 pairs also clustered adjacent to each other on the maximum-likelihood phylogenetic tree (Fig. S4). It is worth mentioning that among the 14 pairs categorized as ‘same strain’, three pairs displayed considerably higher mutation rates compared to previously reported rates of 1.22e−6 to 3.30e−6 base substitutions per nucleotide per year for *S. aureus* [56–63]. Notably, the pair isolated from host 5728 exhibited a mutation rate of 2.3×10^−5^ mutations per nucleotide per year. Furthermore, our analysis revealed that there was no genomic clustering based on the order of clinical isolate collection of the pairs and the host’s CRS phenotype or asthma status (Fig. 1a).

### Chromosomally encoded antimicrobial resistance genes and virulence factors are widespread in *S. aureus* sinonasal isolates

Chromosomally encoded antimicrobial resistance (AMR) genes in the *S. aureus* isolates were assessed using the CARD database, revealing a range of 8–21 genes AMR per isolate. Most isolates (67/68) contained 8–13 AMR genes, including *arlR*, *arLR*, *arlS*, *lmrS*, *mepA*, *mepR*, *mgrA*, *norA* and *tet* [38]*,* which were identified in all clinical isolates (Fig. S5). Only one isolate (Host:2911, CI: C295) contained more than 13 AMR genes. Among the 40 clinical isolates classified as being different strain pairs, the *blaZ* beta-lactamase gene was present in 22 of them. The prevalence of chromosomal *blaZ*-positive isolates increased from 9/20 (45%) in the first timepoint different strain group to 13/20 (65%) in the second timepoint different strain group. None of the isolates in the second timepoint of the different strain group contained the *ermC* gene, whereas, in the first timepoint, three isolates were found to carry multiple copies.

In the same strain group isolates, 11/28 (39.2 %) were positive for a chromosomally encoded *BlaZ* beta-lactamase gene. Only one of the same strain pairs gained a chromosomally encoded *BlaZ* gene at the second timepoint (Fig. S5).

The presence of chromosomally encoded virulence factor genes in the *S. aureus* isolates was assessed using the VFDB database, revealing a range of 45–72 (median 57) genes per isolate (Fig. S6). Notably, all clinical isolates contained the serine protease operon sspABC, also known as V8 protease, which has been previously associated with allergic sensitisation to *S. aureus* [70]. Additionally, all isolates had immune evasion-associated factors such as the immunoglobulin-binding protein *sbi*, *adsA*, *lip*, *hly/hla*, *hlgAB*, *hld*, and *geh*. The *isdABCDEFG* operon was present in 67 out of 68 isolates. The *icaABCD* operon, associated with biofilm production, was present in all isolates, but interestingly, two isolates lacked the *icaR* (repressor) gene. Moreover, 61 and 66 isolates contained the *sak* and *scn* virulence factors, respectively, which are prophage encoded [71]. The prevalence of immune evasion factors *chp* (9/20 vs. 18/20) and *sdrE* (9/20 vs. 15/20) increased in the second timepoint different strain group. In contrast, the carriage of *sdrC* (13/20 vs. 7/20) decreased in the second timepoint of different strain group (Fig. S6). No remarkable alterations were observed in the acquisition or loss of virulence factors between the initial and subsequent timepoints of the same strain group.

### Analysis of virulence profile for incoming isolates

To investigate whether gene presence or absence was linked to persistence, a microbial gene presence-absence analysis was performed on the 34 Timepoint T0 isolates using Scoary. No statistically significant differences in gene content were found between the same and different strain isolates at T0 (BH p.adj >0.05). However, the *chp* gene, involved in chemotaxis inhibition, was less prevalent in the persistent same strain group (8/14 same strain vs. 18/20 different strain).

A subsequent microbial gene presence-absence analysis was conducted on the 40 different strain isolates to examine whether the gene content of the second timepoint T1 isolates differed from that of the T0 isolates. Although no statistically significant differences were observed, incoming T1 isolates contained fewer virulence factors than the T0 isolates they replaced, such as staphylococcal enterotoxins M, U, I, N, and G (present in 8/20 T1 isolates compared to 18/20 T0 isolates) and *chp* (9/20 T1 vs 18/20 T0 isolates).

### Same strain SNV prevalence reveals purifying selection and heterogeneous host adaption

In total, 222 SNVs were observed across the 14 same-strain isolates, ranging from two to 69 per isolate. Eight out of 14 pairs had fewer than ten SNVs. Of these 222 SNVs, 148 were in putative coding sequences (CDS), 44 were synonymous SNVs, three were in-frame variants, nine were frameshift variants, four were stop-gained variants, and the remaining 88 were missense-SNVs, yielding a total of 104 non-synonymous SNVs. Only three genes contained SNVs in more than one isolate, namely the ribosomal protein *rpsJ,* the transcription termination factor *clpC*, and the protease ATP-binding subunit *clpX*.

Using an estimated rate of non-synonymous mutation being 4.6 times higher than that of synonymous mutation in *S. aureus* [55], we approximate a dN/dS ratio of 0.51. This is significantly lower than 1 (*P*<0.001 chi-squared test), suggesting that purifying selection is occurring in same-strain isolates in CRS patients.

*S. aureus* can express a repertoire of approximately 20 different microbial surface components recognizing adhesive matrix molecules (MSCRAMMs) [72]. These surface proteins serve multiple functions, including adhering to and invading host cells and tissues, evading immune responses, and facilitating biofilm formation [73]. MSCRAMMs genes commonly harboured SNVs across the same strain pairs, with 11 SNVs occurring in nine distinct MSCRAMM genes in six distinct isolates, of which 6/11 mutations were synonymous. These include variants in the *sdrC* adhesin, fibronectin-binding proteins A and B (*fnbA* and *fnbB*), surface protein G (*sasG*) and iron-regulated surface-determining proteins *isdD*, *isdE* and *isdF*. Other adhesion genes, including Staphylocoagulase *coa*, extracellular adherence protein *eap*/map, and the extracellular matrix binding protein *EbhA* also had SNVs across isolates.

MSCRAMM genes comprised approximately 2 % of the coding sequences of *S. aureus* on average in the 28 same-strain isolates. There was also little difference in codon composition between MSCRAMM and non-MSCRAMM genes, with the MSCRAMM genes codons having a raw ratio of non-synonymous to synonymous nucleotide changes of 3.41 as compared to 3.55 for the rest of the pangenome. Accordingly, the observed MSCRAMM non-synonymous SNV share of 4.8 % (5/104) (*P*=0.09 exact binomial one-sample proportion test) and a total SNV share of 7.4 % (11/148) (*P*=0.02 exact binomial one-sample proportion test) suggests that there is some evidence MSCRAMM genes are variation hotspots in same-strain CRS isolates.

### Structural variants in same strain clinical isolate pairs involve prophages, insertion sequences, MSCRAMM and AMR genes & are not correlated with the number of SNVs

We detected a total of 37 structural variation among the 14 same strain isolates, ranging in size from small collapsed duplications (<10 bp) to the acquisition of a 43 793 bp *hlb*-disrupting Sa3int prophage in a single isolate pair. Only ten structural variations were larger than 100 bp, and all were found in 4/14 clinical isolate pairs, with one strain having five structural variations >100 bp. Notably, no relationship was observed between the number of SNVs and structural variations. The clinical isolate pair from host 5562, which had the second-lowest number of SNVs [3], had five structural variations, while ten strains did not have any structural variation >100 bp, including four strains with >10 SNVs. In addition, five insertion sequence (IS) insertions were identified in three distinct strains, one of which disrupted the *agr* locus (Table 2).

**Table 2.** Mutation rate and structural variation count between same strain isolate pairs

Host ID clinical isolate pair	SNV count (Snippy)	Time between pairs (years)	Time-corrected SNV (SNV/ years between pairs)	Mutation rate (substitutions/site/year)	SV count (Nucdiff)	SV count (Sniffles)
**420**	69	3.512	19.645	7.09×10^−06^	5	5
**1415**	2	0.805	2.483	8.78×10^−07^	1	2
**1676**	3	0.669	4.482	1.54×10^−06^	1	1
**1992**	6	1.589	3.776	1.38×10^−06^	4	3
**3344**	13	0.552	23.554	8.08×10^−06^	0	1
**3997**	22	4.273	5.148	1.91×10^−06^	0	2
**4681**	6	0.516	11.619	4.25×10^−06^	2	1
**4875**	20	2.704	7.396	2.65×10^−06^	7	5
**5047**	18	3.680	4.891	1.81×10^−06^	1	2
**5060**	5	0.677	7.389	2.64×10^−06^	0	0
**5142**	3	1.178	2.547	9.07×10^−07^	0	0
**5519**	10	1.770	5.650	1.99×10^−06^	2	1
**5562**	3	1.553	1.931	6.81×10-^07^	11	8
**5728**	42	0.647	64.958	2.32×10^−05^	3	4

SNV, Single nucleotide variant; SV, structural variation.

**Fig. 2.**
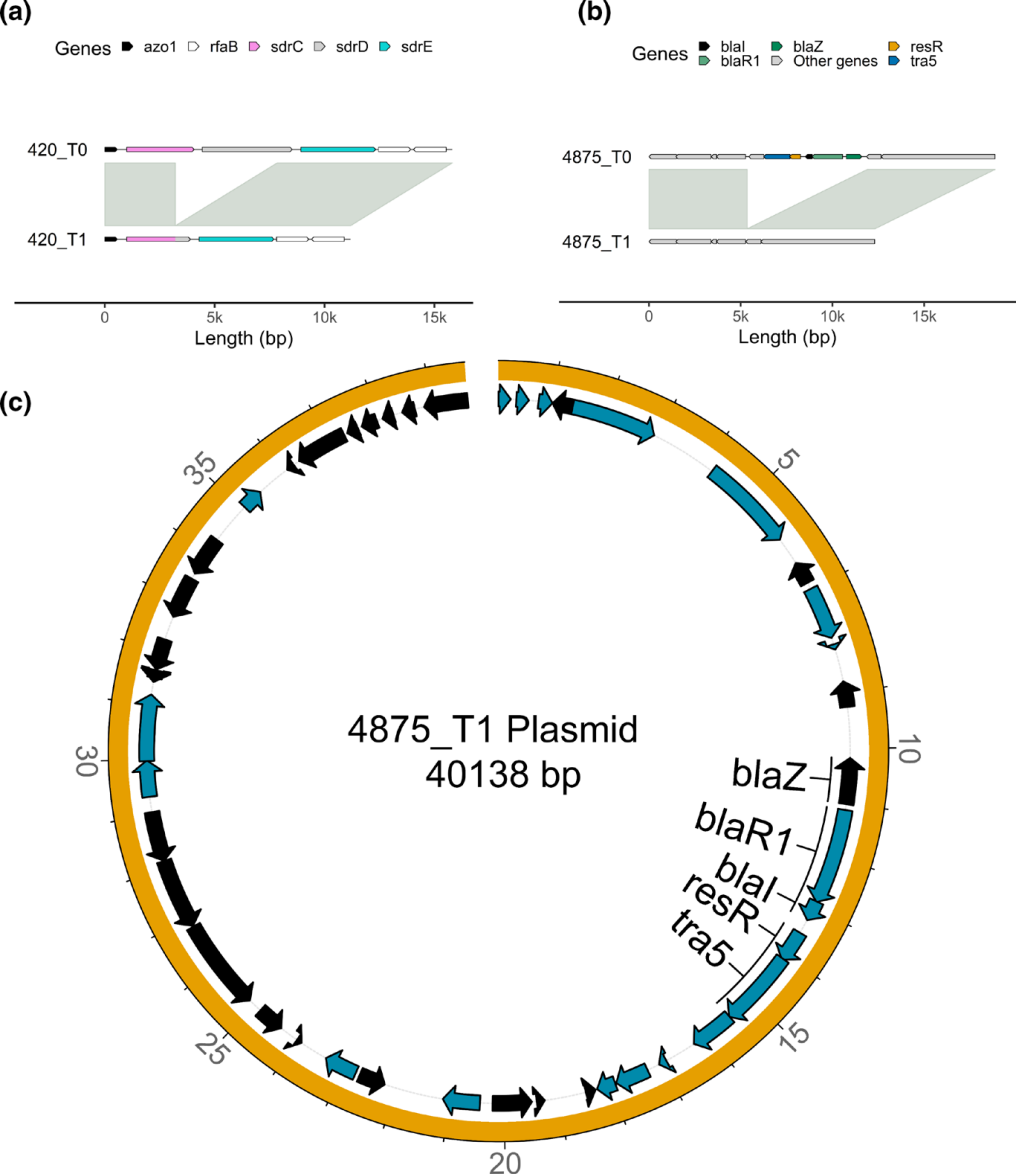
Structural variants identified between same strain longitudinal pairs. (**a**) Alignment of the *sdrCDE* locus between two isolates from the same host (420) at different timepoints. Genes are highlighted in distinct colours, and synteny and sequence similarity are indicated by grey fills connecting the chromosomes. The genomes of the first and second timepoint isolates are shown on top and bottom, respectively. (**b**) Alignment of the β-lactamase locus between two isolates from the same host (4875) at different timepoints. (**c**) Circular plot of the acquired plasmid of the second timepoint isolate of host 4875.

Interestingly, between the same strain clinical isolate pairs obtained from host subject 420, there was a 4638 bp deletion between T0 and T1. This deletion encompassed the cell-wall spanning region, the transmembrane region, and the cytoplasmic domain of the MSCRAMM serine-repeat *sdrC* gene, along with the signal sequence, ligand binding domain and repeat regions in the neighbouring serine-repeat *sdrD* gene as depicted in the coverage and pile-up plot shown in Fig. S7A. This was leading to the recombination of the cell-wall spanning region, the transmembrane region, and the cytoplasmic domain from the *sdrD* gene with the signal sequence, ligand binding domain, and repeat regions of the sdrC gene (Fig. 2a). Additionally, in this clinical isolate pair, the fibrinogen-binding adhesin *SdrG* had a tandem duplication, and there was a tandem duplication in the extracellular adherence protein *Eap/Map* over time.

Another noteworthy observation was the identification of a large structural variation event between the same strain pair from host 4875. A transposon carrying the *blaZ* locus was lost in the second timepoints isolate (Fig. 2b). Specifically, the second timepoint isolate showed a loss of a transposon carrying the *blaZ* locus in the chromosome while simultaneously acquiring a plasmid containing the same locus (Fig. 2b, c). The coverage and pile-up plot shown in Fig. S7B revealed that the coverage of the *blaZ* locus contained by the plasmid was higher compared to that of the chromosome, likely due to the high plasmid copy number (Fig. S7C). These findings highlight the dynamic nature of virulence and AMR genes that occur in persistent *S. aureus* isolates.

### Plasmid carriage is common, and plasmids often encode beta-lactamase resistance genes

**Fig. 3.**
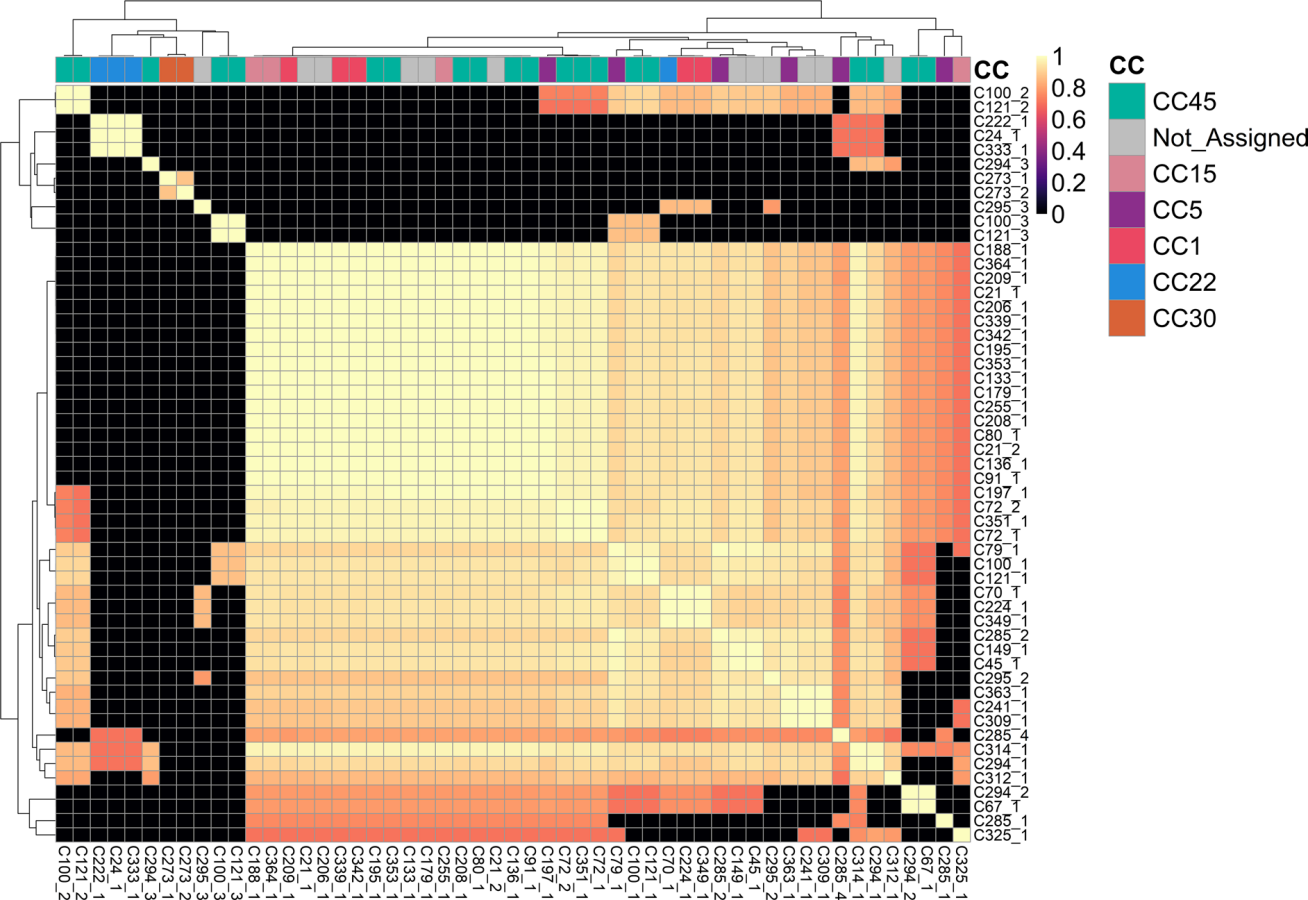
Heatmap displaying the minhash (Mash) distances between the 53 plasmids identified in the 68 clinical isolates. The distances were calculated using mash v2.3 and are represented by a colour gradient. The clonal complex of the clinical isolate from which the plasmid was recovered is indicated at the top of the heatmap.

Staphylococci commonly harbour one or multiple plasmids per cell, each with diverse gene content [74]. Hybrid long and short-read sequencing allowed us to analyse the plasmid content of these isolates and probe their change over time. Fifty-three plasmid contigs were assembled from 41/68 isolates, while the remaining 27 isolates did not carry any plasmids. Fourty-three of fifty-three plasmid contigs were determined to be complete and circularised, while the other ten were putative incomplete contigs. The analysis of the plasmids detected in the 68 clinical isolates revealed a bimodal distribution of Mash distances between each plasmid contig. The analysis revealed a median Mash distance of 0.85 (1st quantile: 0.0, median: 0.85, third quantile: 0.94). Hierarchical clustering of Mash distances revealed two main clusters, with 42/53 plasmid contigs belonging to the larger cluster comprising most of the plasmids in this study. The plasmids in this cluster all had pairwise Mash distances of at least 0.7 with each other (Fig. 3), indicating that the plasmids in this cluster are genetically similar. However, the plasmids in the smaller cluster of 11 contigs showed no overall shared genetic similarity with each other outside groups of two or three, indicating these were rarely found in isolates in this study (Fig. 3). Twenty plasmid contigs were identified in the 28 ‘same strain’ isolate pairs, of which 16 (eight at each timepoint) were present in both timepoints. Plasmid gain was observed between two isolate pairs (subjects 3997 and 4875), where the second timepoint isolates C353 and C294 gained one and two plasmids, respectively. In contrast, plasmid loss was observed in one same strain isolate pair (5047), where the second timepoint isolate C351 lost one plasmid over time.

Twenty-seven of 53 plasmid contigs from 26 distinct isolates carried the beta-lactamase gene *blaZ*.

Of these 27 plasmids, 17/27 were deemed closely related enough to be analysed as the same plasmid based on the empirical thresholds outlined in the Methods (Fig. S8).

Two additional antimicrobial resistance genes were identified in plasmids: erythromycin resistance gene *ErmC* (encoded on a 2473 bp plasmids common to three strains) and the quaternary ammonium compound resistance gene *qacA*, found on one 20 560 bp plasmid.

The number of beta-lactamase encoding plasmids increased from 11 at T0 to 16 at T1, with two same strain isolates acquiring beta-lactamase resistance plasmids. In contrast, three different strain isolates present at T1 replaced isolates at T0 that did not carry a beta-lactamase plasmid, indicating a selection pressure to gain beta-lactamase resistance.

### Plasmid copy numbers increase with time in the same strain group

We found a moderate positive correlation (Spearman’s correlation coefficient R=0.63) between plasmid copy numbers estimated using long and short-read methods (Fig. S9A). The median plasmid copy number estimation was 1.63 times higher in the long-read dataset than in the short-read dataset. Beta-lactamase-carrying plasmids exhibited an even more noticeable difference in copy number estimation between techniques, with a median 2.29 times higher copy number estimate in the long-read dataset. The long-read dataset analysis did not capture four plasmid contigs that were recovered in the short-read dataset (Fig. S9B).

**Fig. 4.**
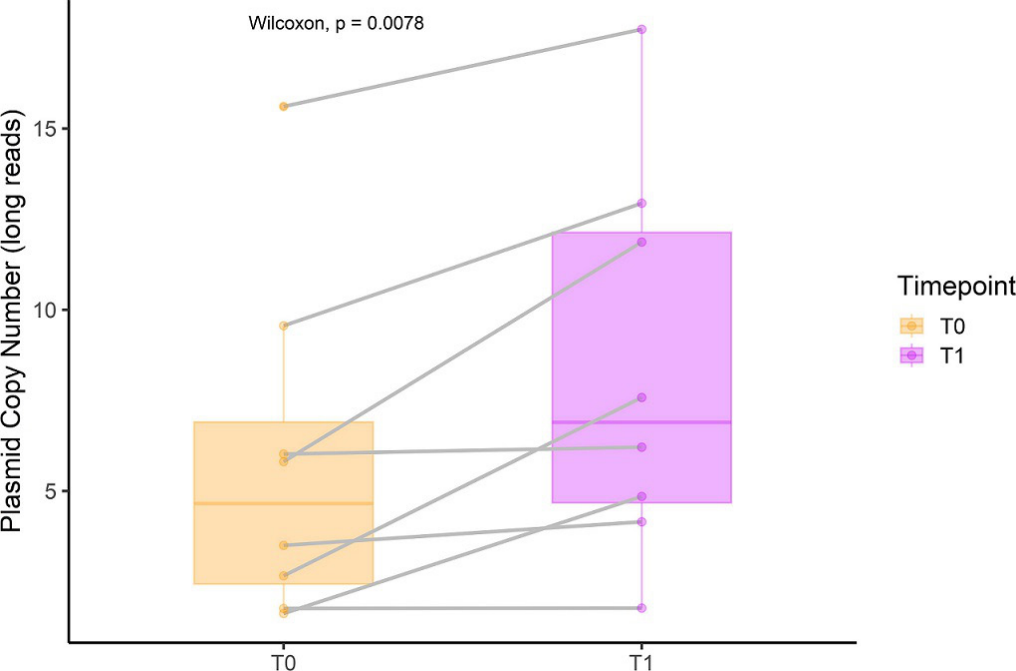
Copy numbers of the conserved plasmids in the ‘same strain’ group (*n*=8) for long-read data. The colour indicates timepoints, and the grey line indicates paired conserved plasmids. The Wilcoxon signed-rank test compared the copy numbers between the two timepoints, with *P*<0.05 considered significant.

We further investigated the stability of plasmid copy numbers in the ‘same strain’ group, focusing on the eight conserved plasmids. We observed a significant increase in the copy number of the conserved plasmids over time (T0: mean copy number 3.29 SE=0.98, T1: mean copy number 6.175 SE=3.87, *P*<0.05) (Fig. 4), with four of the eight conserved isolates being *blaZ* positive. However, we observed no significant difference between the plasmid copy number and timepoint when we examined the short-read data (Fig. S10). It is worth noting that the mean plasmid copy number in the ‘different strain’ group was 4.3 (SE=0.70), providing additional context for our findings.

### Planktonic antibiotic susceptibility remains stable over time

The antibiotic susceptibility of all clinical isolates was tested (*n*=68). Mupirocin appeared to be the most potent, with 85.2 and 67.65% of MIC values below the lowest concentration tested (0.06 mg l^−1^) for the first and second timepoints clinical isolates, respectively. In contrast, erythromycin and clarithromycin had lower susceptibility rates, with over 22% of clinical isolates being resistant to each antibiotic (Table S5). Overall, doxycycline was highly effective at both timepoints, with 97 % of clinical isolates being susceptible. When comparing the first and second timepoints clinical isolates, there was no significant difference in the proportion of resistance between the clinical isolate pairs classified in the different or same strain group (Fig. S11).

### Biofilm antibiotic tolerance increases over time in persistent *S. aureus* strains

**Fig. 5.**
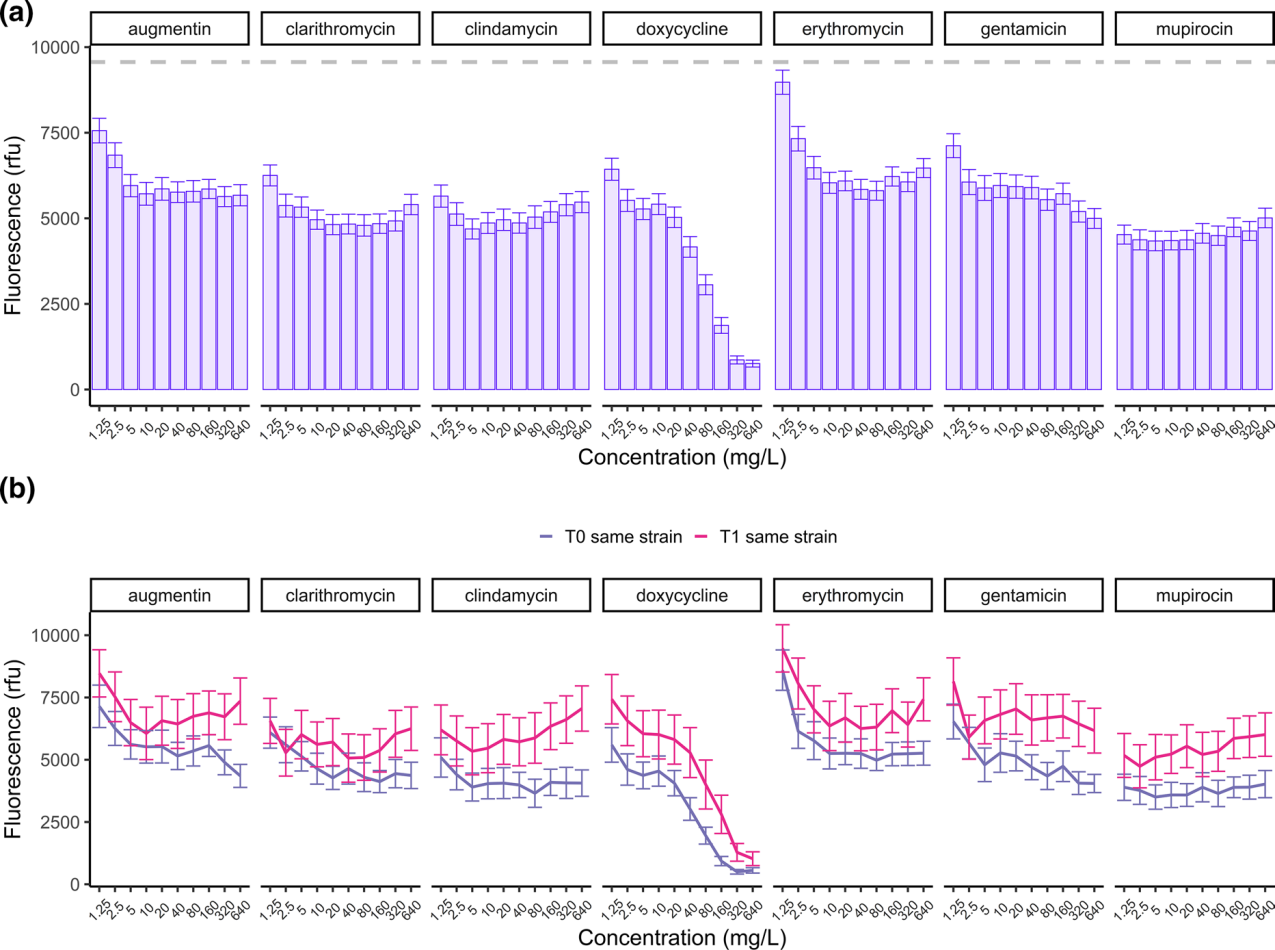
Tolerance of *S. aureus* biofilms to antibiotics. (**a**) Mean biofilm viability after treatment per antibiotic and concentration in relative fluorescence units (rfu) for all 68 clinical isolates. The grey dashed line represents the mean viability of isolates untreated. (**b**) Mean biofilm viability of the first and second clinical isolates pairs classified as the same strain after treatment with increasing concentrations of antibiotics. Error bars represent the standard error of the mean (SEM).

Next, we investigated the antibiotic tolerance of biofilms for all isolates. The viability results after antibiotic treatment were analysed using a GLMM. The model included the following variables: timepoint, antibiotic, logarithmically transformed antibiotic concentration, and same strain-relatedness classification. The antibiotic concentration was log-transformed to facilitate linearisation. The summary statistics of the GLMM results for all effects are provided in Table 3. The biofilm viability data showed high variability in antibiotic tolerance between clinical isolates and antibiotics. Although all antibiotics significantly reduced the biofilm viability (*P*<0.001), their dose-response relationships varied. Except for doxycycline, all antibiotics reached a plateau in their antibiofilm effects at 5 mg l^−1^, reducing biofilm viability by approximately 35 %, and did not eradicate biofilms at the highest concentration (640 mg l^−1^). Notably, mupirocin at the lowest concentration of 1.25 mg l^−1^ showed a reduction of over 50 % in biofilm viability, despite not eradicating the biofilms at 640 mg l^−1^ (Fig. 5a).

**Table 3.** General linear mixed-effect model of biofilm viability

	Fluorescence				
***Predictors***	*Estimates*	*std. Error*	*std. Beta*	*standardized std. Error*	*p*
(**Intercept**)	6238.68	861.46	0.02	0.31	^***^
**group [T1]**	199.56	139.46	0.07	0.05	ns
**same strain [Yes]**	−1097.29	684.77	−0.39	0.25	ns
**CRS phenotype [CRSwNP]**	1006.30	957.54	0.36	0.34	ns
**aspirin sensitivity [1]**	1486.26	905.63	0.53	0.33	ns
**asthma [1]**	−497.92	696.91	−0.18	0.25	ns
**Log(concentration**)	−258.91	14.52	−0.17	0.01	^***^
**antibiotic [clarithromycin]**	−913.07	100.05	−0.33	0.04	^***^
**antibiotic [clindamycin]**	−940.96	100.05	−0.34	0.04	^***^
**antibiotic [doxycycline]**	−2226.13	100.05	−0.80	0.04	^***^
**antibiotic [erythromycin]**	465.69	100.05	0.17	0.04	^***^
**antibiotic [gentamicin]**	−234.94	100.05	−0.08	0.04	^*^
**antibiotic [mupirocin]**	−1526.72	100.05	−0.55	0.04	^***^
**group [T1] Ã— same strain [Yes]**	1487.72	115.88	0.53	0.04	^***^
**group [T1] Ã— CRS phenotype [CRSwNP]**	−166.77	161.03	−0.06	0.06	ns
Random Effects					
**σ^2^**	3403460.07				
**τ_00 id_**	3239247.47				
**N _id_**	34				
**Observations**	4760				
**Marginal R^2^/Conditional R^2^**	0.193/0.587				
*P<0.05 **P<0.01 ***P<0.001					

**Fig. 6.**
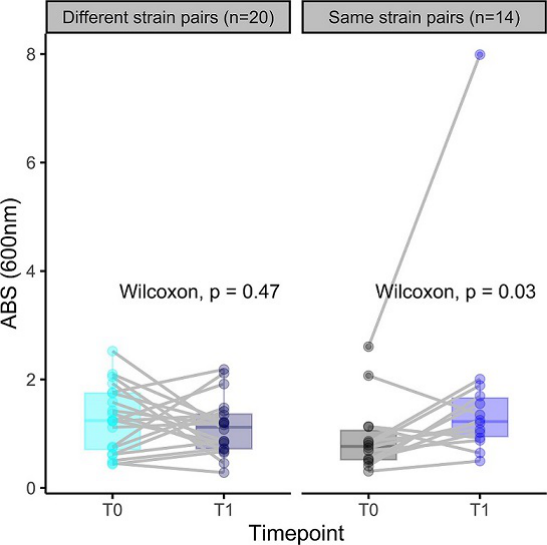
Biomass of *S. aureus* biofilms classified as same strain and different strain using the crystal violet assay. The clinical isolates pairs are connected with a grey line. Paired Wilcoxon test was used to determine the significance between the first and second timepoint. ABS, Absorbance.

Interestingly, we observed a significant increase (*P*<0.001) in antibiotic tolerance of biofilms over time between the first and second isolates classified as ‘same strain’ isolates compared to the first timepoint (Fig. 5b), suggesting that the same strain isolates gained tolerance over time. We then assessed the biofilm biomass using crystal violet staining to investigate the potential relationship between increased antibiotic tolerance and biofilm quantity. We observed a significant increase in the mean biomass of biofilms between the first and second timepoint clinical isolates of the same strain group (paired Wilcoxon signed-rank test, *P*<0.05) (Fig. 6), indicating that the increased biofilm tolerance could be due to increased biofilm production by the same strain isolates over time. A similar trend was seen for the biofilm viability results after 48 h of growth without antibiotic treatment. Specifically, the biofilm fluorescence of the same strain isolates at the first timepoint was significantly lower than that of different strain isolates (*P*<0.05). However, the second timepoint of the same strain group showed a significant increase in biofilm production (*P*<0.01) over time, resulting in no significant difference in biofilm fluorescence between the second timepoint isolates of the same strain and different strain groups (Fig. S12).

Next, we investigated the potential correlation between the number of days of antibiotic usage by CRS patients and the increased biofilm antibiotic tolerance. The most prescribed antibiotic was augmentin, but in terms of total exposure time, doxycycline, followed by sinus/nasal saline irrigation mixed with mupirocin and augmentin, had the most extensive antibiotic exposure in all subjects (Fig. S13). Clinical isolates classified as the same and different strains had a mean exposure of 16.4 days (±7.97) and 15.7 days (±8.39), respectively. However, we did not find a significant relationship between the total number of days of antibiotic exposure and increased biomass between clinical isolates pairs (Spearman correlation coefficient=−0.11, *P*=0.58).

## Discussion

The current study aimed to investigate the persistence of *S. aureus* in the nasal cavity of chronically colonised CRS patients and the related genomic and phenotypic changes over time in a set of longitudinal collections of *S. aureus* clinical isolates. Our hybrid long and short-read sequencing approach allowed us to assemble near-perfect complete genomes and conduct detailed longitudinal genomic analysis. While our study did not identify a specific gene or gene cluster that explains *S. aureus* persistence, persistent isolates often show changes in mobile genetic elements such as plasmids, prophages, and insertion sequences, indicating a potential correlation between the ‘mobilome’ and persistence. The genomic adaptation of persistent isolates was episode-specific, suggesting that each colonisation event may select different adaptations that enable the survival of *S. aureus* in each host. Moreover, the observed increase in biofilm tolerance to antibiotics over time in the same strain isolates, potentially attributed to the growth in biomass, signifies a potential pathoadaptive process by persistent isolates to the sinonasal environment of CRS patients. This finding holds clinical significance and may have implications for treatment strategies.

Although we were unable to determine whether the hosts were persistently or intermittently colonised [75, 76], our study found that out of the 34 clinical isolate pairs, 14 (41 %) were highly related strains based on a two-step approach considering their MLST/PopPUNK clustering and a mutation rate of fewer than 2.5×10^− 5^ mutations/nucleotide/year between isolate pair. Muthukrishnan *et al.* conducted a study on the longitudinal follow-up of *S. aureus* nasal carriage in a healthy population, with a maximum follow-up duration of 3 years. Their findings indicated that colonization by a single strain occurred in the range of 73–77 % of cases. Although this study reported a higher frequency of persistent carriers compared to our results, direct comparisons are challenging due to variations in the time intervals between sample collections in both studies [77]. Additionally, Drilling *et al.* identified that 79 % of recalcitrant CRS patients have a persistent *S. aureus* strain in their paranasal sinuses (mean 98±69 days; range, 12 to 280 days) [11]. However, these results are based on MLST and pulsed-field gel electrophoresis typing, which may overestimate persistent isolates due to less accuracy in discerning strains' genetic relatedness compared to WGS. Thunberg *et al.* reported a lower proportion (20 %) for single-strain long-term colonisation in CRS patients using WGS. This proportion is lower than the 41 % identified in this study. A likely factor contributing to the observed difference could be the extended time lapse of 10 years between the collection of clinical isolate pairs [78]. The longest duration during which we observed a persistent strain was 987 days. It is worth noting that persistence might extend beyond the time frame considered in our study, as there appears to be no distinct correlation between the duration of the sampling period and the turnover of strains in our study.

Furthermore, a considerable proportion of the isolates (35.2 %) did not belong to any known CC based on MLST analysis. Two clinical isolates pairs belonged to the same CC or VLKC while having a SNV counts between pairs exceeding the cutoff threshold of 2.5×10^− 5^ mutations per nucleotide per year and a relatively high structural variation counts between them, highlighting the limitations of a Sequence Typing approach in characterising the genetic similarity of *S. aureus* populations.

The definition of closely related clonal isolates in the literature often employs a threshold-based approach using SNVs divergence. This is typically done by mapping short-read sequences to a reference sequence or calculating core genome SNPs [79, 80]. However, using long-read sequencing technologies enabled us to assemble near-perfect genome assemblies and plasmids instead, which facilitated using the first timepoint isolate as a reference for each longitudinal pair. This revealed that even in low SNV divergent isolate pairs, isolates undergo significant structural changes, such as prophage acquisition, mobile genetic element insertion or loss, and plasmid acquisition, which are difficult or impossible to capture using SNVs only. Additionally, we found no relationship between the number of SNVs and the presence or number of structural variants. Combined with other sequential genomics studies that have revealed similar structural changes in the context of bacteraemia, we suggest that methods that take into account structural variation should complement the genomic adaptation analysis [81, 82]. Interestingly, we observed relatively high mutation rates among three strains classified as the same strain, exceeding the mutational rates reported in previous studies (1.22×10^− 6^ to 3.30×10^− 6^ mutations per nucleotide per year) [56–63]. This discrepancy could be attributed to selective pressure factors such as the frequent use of antibiotics, the chronic inflammatory process observed in CRS patients, or changes in the regulatory network to control virulence [83, 84]. These factors may contribute to increased genetic variability within *S. aureus* populations, potentially leading to higher divergence levels even among closely related strains [85]. Alternatively, it’s conceivable that the host may have been colonized by two closely related yet distinct strains. Another scenario to consider is within-host diversity, where the second isolate may not have descended directly from the first isolate. This could lead to a greater degree of genetic divergence than what would typically be expected.

However, it’s important to approach these interpretations with caution. The absence of mutation rate confidence intervals in our analysis underscores the need for prudence when drawing conclusions about the underlying factors driving these observed mutation rate variations.

MSCRAMM genes are known to be involved in epithelial adhesion and biofilm formation [86, 87]. While a single gene or gene cluster was not found to be indicative of colonisation, our study in CRS patients provides limited evidence for convergent evolution during *S. aureus* nasal carriage. Overall, we observe evidence of purifying selection during *S. aureus* nasal carriage in CRS, with a dN/dS ratio of 0.51, which is similar to the 0.55 reported by Golubchik *et al.* in a study of asymptomatic nasal carriers, indicating selection against changes in coding sequences in same strain isolates over time. We also observed an increased proportion of mutations in genes encoding MSCRAMMs as compared to the rest of the genome. These findings are consistent with a previous study by Golubchik *et al.*, who reported similar trends in their investigation of healthy carriers [55], implying that selection pressure might act on the MSCRAMM genes in chronic colonisation in CRS. We also found that persistent strains had both small and structural MSCRAMM gene variants over time, suggesting that once colonisation has occurred, persistent strains may attenuate their virulence profiles by adaptive evolution over time [88]. While polymorphisms in genes encoding proteins are associated with specific lineages rather than specific hosts [89], it is reasonable to anticipate the occurrence of SNVs in the *S. aureus* genome during host adaptation, especially in pathways related to immune evasion or host-binding proteins [90–92]. Detailed analysis of the *sdr* locus deletion in 4875 revealed recombination of the folding domains from the *sdrC* gene and the wall-spanning and sort domain of the *sdrD* gene, suggesting that intra-host surface adhesin modulation can occur. To our knowledge, such recombination has not been previously reported in *S. aureus *. While it is known that serine-aspartate repeat MSCRAMM proteins are variable and contribute to biofilm formation [93, 94], more work needs to be done to characterise the relationship between divergent serine-aspartate repeat MSCRAMM proteins and their relationship to within-host adaptation.

We employed the Nanopore long-read Rapid Barcoding Kit library preparations, which have been demonstrated to retrieve small plasmids effectively [95]. In line with previous literature, our study found that *S. aureus* isolated from the sinonasal cavity of CRS patients commonly carried one or more plasmids, regardless of their persistence. Furthermore, we observed a high level of similarity among the detected plasmids [96]. These observations emphasize the significance of gene transfer mechanisms of plasmids in *S. aureus *, enabling the exchange of genetic determinants and creating a shared pool of genetic material [97]. While our sample size was insufficient to establish a definitive association between plasmid carriage and lineage [98], we confirmed that these plasmids commonly contained the beta-lactamase gene *blaZ*, which has been frequently found in *S. aureus* strains since the advent of penicillin [99]. Furthermore, our results suggest that *blaZ* encoding plasmids become more prevalent over time, but given the limited sample size, cautious interpretation is warranted.

Overall, our study revealed a trend of higher plasmid copy numbers in the long-read dataset compared to the short-read data, consistent with the findings of Wick *et al.* [95]. We speculate that the discrepancy in copy numbers estimation in the long-read dataset compared to the short may be attributed to the PCR-free nature of the Rapid Barcoding Kit, which could potentially reduce bias compared to PCR-based short-read methods. However, this hypothesis requires further investigation, particularly as long-read sequencing becomes more commonly used, as there is scant literature on the impact of different library preparations on plasmid copy number estimation. Although limited knowledge exists regarding the fitness cost of carriage and copy number of plasmids for *S. aureus *, our study observed an increase in the copy number of conserved plasmids over time in the long-read dataset for the persistent isolates not in the short-read dataset. Plasmids exhibit diverse replication systems, including components for replication initiation and mechanisms for controlling replication [100, 101]. In the absence of selective pressure, the costs of carrying plasmids are expected to outweigh the benefits, resulting in the outcompeting of plasmid-lacking clones or downregulation of plasmid replication within a few generations [102]. However, under selective pressure, such as after antibiotic treatment, the opposite scenario may occur. The observed phenomenon of increased plasmid copy numbers in conserved plasmids could potentially be attributed to the frequent exposure to antibiotics in difficult-to-treat CRS patients. Additionally, the gain of three plasmids in the same strain group suggests a selective pressure for plasmid-encoded traits, which often include antibiotic resistance genes. However, it is important to consider the magnitude of the observed change. Although the mean plasmid copy number approximately doubles, this increase is relatively small in scale, and it remains unclear if it holds biological relevance.

Consistent with previous studies, we observed a high prevalence of macrolide resistance in our set of clinical isolates from CRS patients [103]. Additionally, our findings are consistent with previous studies that have shown a significant decrease in the effectiveness of antibiotics against *S. aureus* biofilms compared to their planktonic counterparts [10]. Only doxycycline was found to have a strong ability to reduce biofilms to near eradication. However, this was only at concentrations exceeding the therapeutic window in humans. These results suggest that antibiotics alone may not be sufficient for eradicating *S. aureus* biofilms in the sinuses of long-term colonized CRS patients, as biofilms are a common feature in the sinuses of CRS patients [104, 105]. Our finding of a frequent persistence of a single *S. aureus* strain in CRS patients is further evidence that the use of topical and systemic antibiotics alone may not be sufficient to eradicate the bacteria. However, we observed a substantial reduction of *S. aureus* biofilms for mupirocin in concentrations achievable when applied topically in saline-based irrigations [106]. Therefore, saline nasal irrigation mixed with mupirocin could play a role in the peri-operative phase of functional endoscopic sinus surgery of CRS patients by reducing the *S. aureus* biofilm, which has been correlated with delayed wound healing and poor post-surgical outcomes [7, 107].

Pathoadaptation of persistent colonisers in (chronic) inflammatory conditions has been described for pathogens such as *S. aureus* and *Pseudomonas aeruginosa* [88, 108]. A surprising finding in this study was the significantly increased biofilm antibiotic tolerance over time of the *S. aureus* strains that are persistent. This increased tolerance was correlated with an increase in the biomass of biofilms of the persistent isolates. The biofilm production and viability in persistent clinical isolates were lower compared to the non-persistent strains at the first timepoint. This suggests that clinical isolates with attenuated biofilm production capacities are more likely to persist in the niche. It can be postulated that the observed increased antibiotic tolerance in those persistent strains over time assists them in their host adaptation, making them well-equipped to occupy and dominate the sinonasal microenvironment of CRS patients, which are frequently exposed to antibiotics. However, it is essential to note that the sinonasal cavity of humans is a relatively low-nutrient environment for bacteria, and high biofilm production might bring a fitness cost [109]. Although our findings indicate an absence of correlation between overall antibiotic exposure and biomass, it is important to note that our limited sample size could contribute to this outcome. It is plausible that the heightened adaptation for increased biofilm production might occur particularly during disease exacerbation, characterised by a substantial bacterial load in the sinuses and exposure to antibiotics. Increased biomass may present a fitness cost during periods between exacerbations, allowing strains with less biofilm production to take over the niche. Our data on non-persistent strains did not show a reduction in biofilm production between the first and second strains. However, the exact timepoint of strain change was not known. In addition, biofilm communities of microorganisms differ from free-floating planktonic microorganisms in various aspects, as they possess protective properties which are regulated via a multitude of mechanisms [110]. Consequently, the substantial increase in biomass observed in biofilms may be attributed to other adaptive mechanisms that, as a side effect, lead to enhanced antibiotic tolerance.

Various mechanisms have been postulated to contribute to biofilm-based antibiotic tolerance of bacteria and the production of extracellular polymeric substances [10, 111]. Although we observed episode-specific mutations in the persistent isolates, we noted that genes involved in adhesion and biofilm formation were frequently affected, suggesting that the accumulation of mutations in different genes can lead to similar phenotypic adaptations.

## Limitations

The findings of this study must be seen in the light of some limitations. Since the study was limited to *S. aureus* clinical isolates from patients suffering from CRS, it was not possible to compare the results to longitudinal clinical isolates from carriers. Longitudinal clinical isolates from carriers with extended follow-up are hard to obtain. Notwithstanding this limitation, this study offers some insight into the genomic and phenotypical adaption of *S. aureus* in the sinuses of CRS patients. Furthermore, the scope of the genomic analysis in this study was limited due to the low sample size. The genomic complexity of *S. aureus* does not lend itself to genome-wide association studies in low sample size populations. In our study, we sequenced a single colony per timepoint rather than multiple colonies or the entire primary swab. Therefore, additional uncontrolled factors are the possibility of co-colonization or intra-host diversity of *S. aureus* at a single timepoint. Previous studies have reported the presence of multiple strains of *S. aureus* in nasal carriers. Votintseva *et al.* observed that approximately 5% of nasal carriers carry more than one strain of *S. aureus* simultaneously [112]. These findings underscore the intricate dynamics of *S. aureus* colonization and the possibility of coexistence of different strains within the same host. To capture the presence of multiple strains, collecting multiple samples from each timepoint can be a valuable approach. However, even with this approach, there is still a possibility of missing certain strain combinations. Thunberg *et al.* reported a case where two different strains were found in a single host, one isolated from the sinus and the other from the nasal passage of CRS patients [113]. This highlights the challenges in accurately assessing and characterizing the complete extent of multiple-strain colonization, especially when relying on conventional culturing methods. Nonetheless, we acknowledge that the presence of multiple *S. aureus* strains in the nasal cavities of patients can influence our results impacting the observed genetic diversity and strain persistence rate.

Furthermore, the single colony approach may limit our ability to capture the full extent of intra-host diversity within a host at a single timepoint as studies have uncovered within host *S *. *aureus* genetic heterogeneity [114, 115]. While this approach provides valuable insights into the dynamics of the dominant strain over time, it may not fully capture the diversity of subpopulations or minor variants within the host which often lead to adaption to the specific conditions in a given host [85]. Moreover, it does not allow us to fully differentiate between within-host variation and within-host evolution of the same strain group, which may potentially lead to an overestimation of within-host evolution. However, even considering the potential influence of within-host variation, our study still provides valuable insights into the dynamics of a within-host dynamics of a single strain over time.

A natural progression of this work is to analyse the genome of specific clinical isolate pairs and all the in-between clinical isolates to identify a genomic target that might be involved in the phenotypical adaptation.

## Conclusion

Our findings provide insights into *S. aureus* persistence in difficult-to-treat CRS and highlight the resilience of bacterial biofilms. Our results shed light on the genomic and phenotypic changes associated with the persistence of *S. aureus* in chronically colonised CRS patients. Further studies are needed to understand the mechanisms underlying these adaptations and their potential survival benefit to identify potential targets for developing new eradication strategies.

## Supplementary Data

Supplementary material 1

## References

[1] Fokkens WJ, Lund VJ, Hopkins C, Hellings PW, Kern R (2020). European position paper on rhinosinusitis and nasal polyps 2020. Rhinology.

[2] Hastan D, Fokkens WJ, Bachert C, Newson RB, Bislimovska J (2011). Chronic rhinosinusitis in Europe--an underestimated disease. A GA(2)LEN study. Allergy.

[3] Hopkins C (2019). Chronic rhinosinusitis with nasal polyps. N Engl J Med.

[4] Okifo O, Ray A, Gudis DA (2022). The microbiology of acute exacerbations in chronic rhinosinusitis - a systematic review. Front Cell Infect Microbiol.

[5] Vickery TW, Ramakrishnan VR, Suh JD (2019). The role of *Staphylococcus aureus* in patients with chronic sinusitis and nasal polyposis. Curr Allergy Asthma Rep.

[6] Hoggard M, Wagner Mackenzie B, Jain R, Taylor MW, Biswas K (2017). Chronic rhinosinusitis and the evolving understanding of microbial ecology in chronic inflammatory mucosal disease. Clin Microbiol Rev.

[7] Psaltis AJ, Weitzel EK, Ha KR, Wormald PJ (2008). The effect of bacterial biofilms on post-sinus surgical outcomes. Am J Rhinol.

[8] Singhal D, Foreman A, Jervis-Bardy J, Wormald PJ (2011). *Staphylococcus aureus* biofilms: Nemesis of endoscopic sinus surgery. Laryngoscope.

[9] Barshak MB, Durand ML (2017). The role of infection and antibiotics in chronic rhinosinusitis. Laryngoscope Investig Otolaryngol.

[10] Hall CW, Mah TF (2017). Molecular mechanisms of biofilm-based antibiotic resistance and tolerance in pathogenic bacteria. FEMS Microbiol Rev.

[11] Drilling A, Coombs GW, Tan H, Pearson JC, Boase S (2014). Cousins, siblings, or copies: the genomics of recurrent *Staphylococcus aureus* infections in chronic rhinosinusitis. Int Forum Allergy Rhinol.

[12] Bachert C, Humbert M, Hanania NA, Zhang N, Holgate S (2020). *Staphylococcus aureus* and its IgE-inducing enterotoxins in asthma: current knowledge. Eur Respir J.

[13] Schechner V, Temkin E, Harbarth S, Carmeli Y, Schwaber MJ (2013). Epidemiological interpretation of studies examining the effect of antibiotic usage on resistance. Clin Microbiol Rev.

[14] Shaghayegh G, Cooksley C, Bouras GS, Panchatcharam BS, Idrizi R (2023). Chronic rhinosinusitis patients display an aberrant immune cell localization with enhanced *S aureus* biofilm metabolic activity and biomass. J Allergy Clin Immunol.

[15] Mölder F, Jablonski KP, Letcher B, Hall MB, Tomkins-Tinch CH (2021). Sustainable data analysis with snakemake. F1000Res.

[16] Roach MJ, Pierce-Ward NT, Suchecki R, Mallawaarachchi V, Papudeshi B (2022). Ten simple rules and a template for creating workflows-as-applications. PLoS Comput Biol.

[17] Bouras G hybracter hybracter. https://github.com/gbouras13/hybracter.

[18] Wick RR, Judd LM, Holt KE (2023). Assembling the perfect bacterial genome using Oxford Nanopore and Illumina sequencing. PLoS Comput Biol.

[19] Hall M (2022). Rasusa: randomly subsample sequencing reads to a specified coverage. JOSS.

[20] Wick RR Filtlong Filtlong. https://github.com/rrwick/Filtlong.

[21] Wick RR Porechop Porechop. https://github.com/rrwick/Porechop.

[22] Chen S, Zhou Y, Chen Y, Gu J (2018). fastp: an ultra-fast all-in-one FASTQ preprocessor. Bioinformatics.

[23] Kolmogorov M, Yuan J, Lin Y, Pevzner PA (2019). Assembly of long, error-prone reads using repeat graphs. Nat Biotechnol.

[24] ONT (2022). Medaka. https://github.com/nanoporetech/medaka.

[25] Bouras G dnaapler dnaapler. https://github.com/gbouras13/dnaapler.

[26] Wick RR, Holt KE (2022). Polypolish: short-read polishing of long-read bacterial genome assemblies. PLoS Comput Biol.

[27] Zimin AV, Salzberg SL (2020). The genome polishing tool POLCA makes fast and accurate corrections in genome assemblies. PLoS Comput Biol.

[28] Bouras G, Sheppard AE, Mallawaarachchi V, Vreugde S (2023). Plassembler: an automated bacterial plasmid assembly tool. Bioinformatics.

[29] De Coster W, D’Hert S, Schultz DT, Cruts M, Van Broeckhoven C (2018). NanoPack: visualizing and processing long-read sequencing data. Bioinformatics.

[30] Li H (2018). Minimap2: pairwise alignment for nucleotide sequences. Bioinformatics.

[31] Li H (2013). Aligning sequence reads, clone sequences and assembly Contigs with BWA-MEM. arXiv preprint arXiv.

[32] Wick RR, Judd LM, Gorrie CL, Holt KE (2017). Unicycler: resolving bacterial genome assemblies from short and long sequencing reads. PLoS Comput Biol.

[33] Schwengers O, Jelonek L, Dieckmann MA, Beyvers S, Blom J (2021). Bakta: rapid and standardized annotation of bacterial genomes via alignment-free sequence identification. Microb Genom.

[34] Seemann T mlst mlst. https://github.com/tseemann/mlst.

[35] Jolley KA, Bray JE, Maiden MCJ (2018). Open-access bacterial population genomics: BIGSdb software, the PubMLST.org website and their applications. Wellcome Open Res.

[36] Lees JA, Harris SR, Tonkin-Hill G, Gladstone RA, Lo SW (2019). Fast and flexible bacterial genomic epidemiology with PopPUNK. Genome Res.

[37] Petit RA, Read TD (2018). *Staphylococcus aureus* viewed from the perspective of 40,000+ genomes. PeerJ.

[38] Xu S, Li L, Luo X, Chen M, Tang W (2022). *Ggtree*: a serialized data object for visualization of a phylogenetic tree and annotation data. iMeta.

[39] Tonkin-Hill G, MacAlasdair N, Ruis C, Weimann A, Horesh G (2020). Producing polished prokaryotic pangenomes with the Panaroo pipeline. Genome Biol.

[40] Minh BQ, Schmidt HA, Chernomor O, Schrempf D, Woodhams MD (2020). IQ-TREE 2: new models and efficient methods for phylogenetic inference in the genomic era. Mol Biol Evol.

[41] Seemann T Abricate Abricate. https://github.com/tseemann/abricate.

[42] Jia B, Raphenya AR, Alcock B, Waglechner N, Guo P (2017). CARD 2017: expansion and model-centric curation of the comprehensive antibiotic resistance database. Nucleic Acids Res.

[43] Liu B, Zheng D, Jin Q, Chen L, Yang J (2019). VFDB 2019: a comparative pathogenomic platform with an interactive web interface. Nucleic Acids Res.

[44] Brynildsrud O, Bohlin J, Scheffer L, Eldholm V (2016). Rapid scoring of genes in microbial pan-genome-wide association studies with scoary. Genome Biol.

[45] Seemann T (2015). Snippy: fast bacterial variant calling from NGS reads. https://github.com/tseemann/snippy.

[46] Khelik K, Lagesen K, Sandve GK, Rognes T, Nederbragt AJ (2017). NucDiff: in-depth characterization and annotation of differences between two sets of DNA sequences. BMC Bioinformatics.

[47] Sedlazeck FJ, Rescheneder P, Smolka M, Fang H, Nattestad M (2018). Accurate detection of complex structural variations using single-molecule sequencing. Nat Methods.

[48] Danecek P, Bonfield JK, Liddle J, Marshall J, Ohan V (2021). Twelve years of SAMtools and BCFtools. Gigascience.

[49] Hahne F, Ivanek R, Mathé E, Davis S (2016). Statistical Genomics: Methods and Protocols.

[50] Ankenbrand Th (2022). gggenomes: a grammar of graphics for comparative genomics.

[51] Robertson J, Nash JHE (2018). MOB-suite: software tools for clustering, reconstruction and typing of plasmids from draft assemblies. Microb Genom.

[52] Ondov BD, Treangen TJ, Melsted P, Mallonee AB, Bergman NH (2016). Mash: fast genome and metagenome distance estimation using MinHash. Genome Biol.

[53] Hawkey J, Wyres KL, Judd LM, Harshegyi T, Blakeway L (2022). ESBL plasmids in *Klebsiella pneumoniae*: diversity, transmission and contribution to infection burden in the hospital setting. Genome Med.

[54] Rocha EPC, Smith JM, Hurst LD, Holden MTG, Cooper JE (2006). Comparisons of dN/dS are time dependent for closely related bacterial genomes. J Theor Biol.

[55] Golubchik T, Batty EM, Miller RR, Farr H, Young BC (2013). Within-host evolution of *Staphylococcus aureus* during asymptomatic carriage. PLoS One.

[56] Holden MTG, Hsu L-Y, Kurt K, Weinert LA, Mather AE (2013). A genomic portrait of the emergence, evolution, and global spread of a methicillin-resistant *Staphylococcus aureus* pandemic. Genome Res.

[57] McAdam PR, Templeton KE, Edwards GF, Holden MTG, Feil EJ (2012). Molecular tracing of the emergence, adaptation, and transmission of hospital-associated methicillin-resistant *Staphylococcus aureus*. Proc Natl Acad Sci U S A.

[58] Nübel U, Dordel J, Kurt K, Strommenger B, Westh H (2010). A timescale for evolution, population expansion, and spatial spread of an emerging clone of methicillin-resistant *Staphylococcus aureus*. PLoS Pathog.

[59] Ward MJ, Gibbons CL, McAdam PR, van Bunnik BAD, Girvan EK (2014). Time-scaled evolutionary analysis of the transmission and antibiotic resistance dynamics of *Staphylococcus aureus* clonal complex 398. Appl Environ Microbiol.

[60] Uhlemann A-C, Dordel J, Knox JR, Raven KE, Parkhill J (2014). Molecular tracing of the emergence, diversification, and transmission of S. aureus sequence type 8 in a New York community. Proc Natl Acad Sci U S A.

[61] Baines SL, Holt KE, Schultz MB, Seemann T, Howden BO (2015). Convergent adaptation in the dominant global hospital clone ST239 of methicillin-resistant *Staphylococcus aureus*. mBio.

[62] Harris SR, Feil EJ, Holden MTG, Quail MA, Nickerson EK (2010). Evolution of MRSA during hospital transmission and intercontinental spread. Science.

[63] Szafrańska AK, Junker V, Steglich M, Nübel U (2019). Rapid cell division of *Staphylococcus aureus* during colonization of the human nose. BMC Genomics.

[64] Wiegand I, Hilpert K, Hancock REW (2008). Agar and broth dilution methods to determine the minimal inhibitory concentration (MIC) of antimicrobial substances. Nat Protoc.

[65] Mah TF (2014). Establishing the minimal bactericidal concentration of an antimicrobial agent for planktonic cells (MBC-P) and biofilm cells (MBC-B). J Vis Exp.

[66] Pettit RK, Weber CA, Kean MJ, Hoffmann H, Pettit GR (2005). Microplate Alamar blue assay for Staphylococcus epidermidis biofilm susceptibility testing. Antimicrob Agents Chemother.

[67] Stiefel P, Rosenberg U, Schneider J, Mauerhofer S, Maniura-Weber K (2016). Is biofilm removal properly assessed? Comparison of different quantification methods in a 96-well plate system. Appl Microbiol Biotechnol.

[68] R Core Team (2017). R: A Language and Environment for Statistical Computing.

[69] Key FM, Khadka VD, Romo-González C, Blake KJ, Deng L (2023). On-person adaptive evolution of *Staphylococcus aureus* during treatment for atopic dermatitis. Cell Host Microbe.

[70] Krysko O, Teufelberger A, Van Nevel S, Krysko DV, Bachert C (2019). Protease/antiprotease network in allergy: the role of *Staphylococcus aureus* protease-like proteins. Allergy.

[71] Nepal R, Houtak G, Shaghayegh G, Bouras G, Shearwin K (2021). Prophages encoding human immune evasion cluster genes are enriched in *Staphylococcus aureus* isolated from chronic rhinosinusitis patients with nasal polyps. Microb Genom.

[72] Mazmanian SK, Liu G, Ton-That H, Schneewind O (1999). *Staphylococcus aureus* sortase, an enzyme that anchors surface proteins to the cell wall. Science.

[73] Foster TJ, Geoghegan JA, Ganesh VK, Höök M (2014). Adhesion, invasion and evasion: the many functions of the surface proteins of *Staphylococcus aureus*. Nat Rev Microbiol.

[74] Malachowa N, DeLeo FR (2010). Mobile genetic elements of *Staphylococcus aureus*. Cell Mol Life Sci.

[75] Sollid JUE, Furberg AS, Hanssen AM, Johannessen M (2014). *Staphylococcus aureus*: determinants of human carriage. Infect Genet Evol.

[76] Price JR, Cole K, Bexley A, Kostiou V, Eyre DW (2017). Transmission of *Staphylococcus aureus* between health-care workers, the environment, and patients in an intensive care unit: a longitudinal cohort study based on whole-genome sequencing. Lancet Infect Dis.

[77] Muthukrishnan G, Lamers RP, Ellis A, Paramanandam V, Persaud AB (2013). Longitudinal genetic analyses of *Staphylococcus aureus* nasal carriage dynamics in a diverse population. BMC Infect Dis.

[78] Thunberg U, Hugosson S, Ehricht R, Monecke S, Müller E (2021). Long-term sinonasal carriage of *Staphylococcus aureus* and anti-staphylococcal humoral immune response in patients with chronic rhinosinusitis. Microorganisms.

[79] Lagos AC, Sundqvist M, Dyrkell F, Stegger M, Söderquist B (2022). Evaluation of within-host evolution of methicillin-resistant *Staphylococcus aureus* (MRSA) by comparing cgMLST and SNP analysis approaches. Sci Rep.

[80] Coll F, Raven KE, Knight GM, Blane B, Harrison EM (2020). Definition of a genetic relatedness cutoff to exclude recent transmission of meticillin-resistant *Staphylococcus aureus*: a genomic epidemiology analysis. Lancet Microbe.

[81] Giulieri SG, Baines SL, Guerillot R, Seemann T, Gonçalves da Silva A (2018). Genomic exploration of sequential clinical isolates reveals a distinctive molecular signature of persistent *Staphylococcus aureus* bacteraemia. Genome Med.

[82] Giulieri SG, Guérillot R, Duchene S, Hachani A, Daniel D (2022). Niche-specific genome degradation and CONVERGENT evolution shaping *Staphylococcus aureus* adaptation during severe infections. Elife.

[83] Altman DR, Sullivan MJ, Chacko KI, Balasubramanian D, Pak TR (2018). Genome plasticity of *agr*-defective *Staphylococcus aureus* during clinical infection. Infect Immun.

[84] Jenul C, Horswill AR (2019). Regulation of *Staphylococcus aureus* virulence. Microbiol Spectr.

[85] Didelot X, Walker AS, Peto TE, Crook DW, Wilson DJ (2016). Within-host evolution of bacterial pathogens. Nat Rev Microbiol.

[86] Foster TJ (2019). The MSCRAMM family of cell-wall-anchored surface proteins of gram-positive cocci. Trends Microbiol.

[87] Raafat D, Otto M, Reppschlager K, Iqbal J, Holtfreter S (2019). Fighting *Staphylococcus aureus* biofilms with monoclonal antibodies. Trends Microbiol.

[88] Howden BP, Giulieri SG, Wong Fok Lung T, Baines SL, Sharkey LK (2023). *Staphylococcus aureus* host interactions and adaptation. Nat Rev Microbiol.

[89] McCarthy AJ, Lindsay JA (2013). *Staphylococcus aureus* innate immune evasion is lineage-specific: a bioinfomatics study. Infect Genet Evol.

[90] Thammavongsa V, Kim HK, Missiakas D, Schneewind O (2015). Staphylococcal manipulation of host immune responses. Nat Rev Microbiol.

[91] Haim MS, Zaheer R, Bharat A, Di Gregorio S, Di Conza J (2021). Comparative genomics of ST5 and ST30 methicillin-resistant *Staphylococcus aureus* sequential isolates recovered from paediatric patients with cystic fibrosis. Microb Genom.

[92] Solis N, Parker BL, Kwong SM, Robinson G, Firth N (2014). *Staphylococcus aureus* surface proteins involved in adaptation to oxacillin identified using a novel cell shaving approach. J Proteome Res.

[93] Ajayi C, Åberg E, Askarian F, Sollid JUE, Johannessen M (2018). Genetic variability in the sdrD gene in *Staphylococcus aureus* from healthy nasal carriers. BMC Microbiol.

[94] Barbu EM, Mackenzie C, Foster TJ, Höök M (2014). SdrC induces staphylococcal biofilm formation through a homophilic interaction. Mol Microbiol.

[95] Wick RR, Judd LM, Wyres KL, Holt KE (2021). Recovery of small plasmid sequences via Oxford Nanopore sequencing. Microb Genom.

[96] Shearer JES, Wireman J, Hostetler J, Forberger H, Borman J (2011). Major families of multiresistant plasmids from geographically and epidemiologically diverse staphylococci. G3.

[97] Firth N, Jensen SO, Kwong SM, Skurray RA, Ramsay JP (2018). Staphylococcal plasmids, transposable and integrative elements. Microbiol Spectr.

[98] McCarthy AJ, Lindsay JA (2012). The distribution of plasmids that carry virulence and resistance genes in *Staphylococcus aureus* is lineage associated. BMC Microbiol.

[99] Turner NA, Sharma-Kuinkel BK, Maskarinec SA, Eichenberger EM, Shah PP (2019). Methicillin-resistant *Staphylococcus aureus*: an overview of basic and clinical research. Nat Rev Microbiol.

[100] Brantl S (2014). Plasmid replication control by antisense RNAs. Microbiol Spectr.

[101] Ruiz-Masó JA, MachóN C, Bordanaba-Ruiseco L, Espinosa M, Coll M (2015). Plasmid rolling-circle replication. Microbiol Spectr.

[102] San Millan A, MacLean RC (2017). Fitness costs of plasmids: a limit to plasmid transmission. Microbiol Spectr.

[103] Bhattacharyya N, Kepnes LJ (2008). Assessment of trends in antimicrobial resistance in chronic rhinosinusitis. Ann Otol Rhinol Laryngol.

[104] Singhal D, Psaltis AJ, Foreman A, Wormald PJ (2010). The impact of biofilms on outcomes after endoscopic sinus surgery. Am J Rhinol Allergy.

[105] Foreman A, Psaltis AJ, Tan LW, Wormald PJ (2010). Characterization of bacterial and fungal biofilms in chronic rhinosinusitis. Allergy Rhinol.

[106] Kim JS, Kwon SH (2016). Mupirocin in the treatment of staphylococcal infections in chronic rhinosinusitis: a meta-analysis. PLoS One.

[107] Percival SL (2017). Importance of biofilm formation in surgical infection. Br J Surg.

[108] Rossi E, La Rosa R, Bartell JA, Marvig RL, Haagensen JAJ (2021). *Pseudomonas aeruginosa* adaptation and evolution in patients with cystic fibrosis. Nat Rev Microbiol.

[109] Krismer B, Liebeke M, Janek D, Nega M, Rautenberg M (2014). Nutrient limitation governs *Staphylococcus aureus* metabolism and niche adaptation in the human nose. PLoS Pathog.

[110] Flemming HC, Wingender J, Szewzyk U, Steinberg P, Rice SA (2016). Biofilms: an emergent form of bacterial life. Nat Rev Microbiol.

[111] Karygianni L, Ren Z, Koo H, Thurnheer T (2020). Biofilm matrixome: extracellular components in structured microbial communities. Trends Microbiol.

[112] Votintseva AA, Miller RR, Fung R, Knox K, Godwin H (2014). Multiple-strain colonization in nasal carriers of *Staphylococcus aureus*. J Clin Microbiol.

[113] Thunberg U, Hugosson S, Ehricht R, Monecke S, Müller E (2021). Long-term sinonasal carriage of *Staphylococcus aureus* and anti-staphylococcal humoral immune response in patients with chronic rhinosinusitis. Microorganisms.

[114] Howden BP, Giulieri SG, Wong Fok Lung T, Baines SL, Sharkey LK (2023). Staphylococcus aureus host interactions and adaptation. Nat Rev Microbiol.

[115] Azarian T, Ridgway JP, Yin Z, David MZ (2019). Long-term intrahost evolution of methicillin resistant *Staphylococcus aureus* among cystic fibrosis patients with respiratory carriage. Front Genet.

