# Supplementary material for: The intra-host evolutionary landscape and pathoadaptation of persistent Staphylococcus aureus in chronic rhinosinusitis
Source: Microb Genom. 2023 Nov 27;9(11):001128. doi: 10.1099/mgen.0.001128 (PMC10711304; doi:10.1099/mgen.0.001128)
Supplement: Supplementary material 1 [file mgen-9-1128-s001.pdf]

Table S1. Biosample accession number for each sample

Biosample accession	Sample id	Host id and timepoint
**SAMN32360832**	C22	420_T0
**SAMN32360833**	C265	1415_T0
**SAMN32360834**	C13	1676_T0
**SAMN32360835**	C80	1992_T0
**SAMN32360836**	C295	2911_T1
**SAMN32360837**	C52	3344_T0
**SAMN32360838**	C9	3997_T0
**SAMN32360839**	C3	4681_T0
**SAMN32360840**	C67	4875_T0
**SAMN32360841**	C72	5047_T0
**SAMN32360842**	C45	5142_T0
**SAMN32360843**	C224	5519_T0
**SAMN32360844**	C222	5562_T0
**SAMN32360845**	C241	5728_T0
**SAMN32360846**	C100	276_T0
**SAMN32360847**	C364	276_T1
**SAMN32360848**	C320	420_T1
**SAMN32360849**	C235	539_T0
**SAMN32360850**	C318	539_T1
**SAMN32360851**	C148	1170_T1
**SAMN32360852**	C79	1170_T0
**SAMN32360853**	C324	1415_T1
**SAMN32360854**	C76	1676_T1
**SAMN32360855**	C208	1992_T1
**SAMN32360856**	C240	2911_T0
**SAMN32360857**	C113	3344_T1
**SAMN32360858**	C195	3357_T1
**SAMN32360859**	C24	3357_T0
**SAMN32360860**	C353	3997_T1
**SAMN32360861**	C121	4009_T0
**SAMN32360862**	C255	4009_T1
**SAMN32360863**	C56	4681_T1
**SAMN32360864**	C188	4747_T1
**SAMN32360865**	C32	4747_T0
**SAMN32360866**	C16	4784_T0
**SAMN32360867**	C70	4784_T1
**SAMN32360868**	C294	4875_T1

**SAMN32360869**	C21	4986_T0
**SAMN32360870**	C273	4986_T1
**SAMN32360871**	C351	5047_T1
**SAMN32360872**	C133	5060_T0
**SAMN32360873**	C179	5060_T1
**SAMN32360874**	C149	5142_T1
**SAMN32360875**	C209	5308_T1
**SAMN32360876**	C91	5308_T0
**SAMN32360877**	C206	5328_T0
**SAMN32360878**	C276	5328_T1
**SAMN32360879**	C136	5390_T0
**SAMN32360880**	C197	5390_T1
**SAMN32360881**	C155	5448_T0
**SAMN32360882**	C339	5448_T1
**SAMN32360883**	C196	5469_T0
**SAMN32360884**	C342	5469_T1
**SAMN32360885**	C183	5485_T0
**SAMN32360886**	C312	5485_T1
**SAMN32360887**	C182	5503_T0
**SAMN32360888**	C233	5503_T1
**SAMN32360889**	C349	5519_T1
**SAMN32360890**	C333	5562_T1
**SAMN32360891**	C245	5631_T0
**SAMN32360892**	C314	5631_T1
**SAMN32360893**	C285	5647_T0
**SAMN32360894**	C355	5647_T1
**SAMN32360895**	C309	5728_T1
**SAMN32360896**	C325	5767_T0
**SAMN32360897**	C363	5767_T1
**SAMN32360898**	C311	5911_T0
**SAMN32360899**	C357	5911_T1

Table S2. MSCRAMM genes

**Gene (Panaroo Cluster)**	**No. isolates with gene**	**Mean size gene (nucleotide)**
isdB	68	1941.62
clfB	68	2668.54
mscL	68	366
isdF	68	969
isdD	68	1077
isdE	68	879
ebhB~~~ebh~~~ebhA	68	17749.25
efb	68	470.65
isdA	68	1057.63
sdrC	68	2838.70
clfA	68	2861.82
isdC	68	684.88
eap	68	1372.21
fnbA	66	2933
sdrD~~~clfA	56	4095.63
sdrE	55	3444.82
fnbB	49	2863.84
cna	42	3002.07
sasG	29	2575.62

Table S3. Metadata characteristics of chronic rhinosinusitis subjects and corresponding clinical isolate collection

Characteristic	CRSwNP	CRSsNP	Total
**Host subjects-N**	29	5	34
**Gender male-N**	12	2	14
**Age -year (± SD)**	51 (± 11)	54 (± 21)	52 (± 15)
**Aspirin sensitivity-N**	6	0	6
**Asthmatic-N**	17	2	19
**Mean time between clinical isolate pair collection- days (± SD)**	565 (±398)	558 (±362)	563 (±387)

CRSwNP, chronic rhinosinusitis with nasal polyps, CRSsNP, chronic rhinosinusitis without nasal polyps.

Table S4. Compilation of metadata characteristics of clinical isolate by relatedness classification and host characteristics

Characteristic	Same strain pairs	Different strain pairs	Total
**Count-N (% of total)**	14 (41%)	20 (59%)	34 (100%)
**Gender of host-Male-N**	5	9	14
**Age of host -year (± SD)**	57 (± 15)	50 (± 16)	52 (± 15)
**Phenotype of host-CRSwNP- N**	14	15	29
**Aspirin sensitivity of host-N**	3	3	6
**Asthma status of host-N**	7	12	19
**Mean time between clinical isolate pair collection- days (± SD)**	629 (±474)	518 (±320)	563(±387)

CRSwNP, chronic rhinosinusitis with nasal polyps.

Table S5. Frequency distribution of *Staphylococcus aureus* isolates' Minimum Inhibitory Concentration (MIC)

Antibiotic	Timepoint *	MIC (%) Concentration (mg/L)												MIC_{50}	MIC_{90}	Resistant (%)
		<0.06	0.06	0.125	0.25	0.5	1	2	4	8	16	32	>32			
Erythromycin	T0	0.00	0.00	0.00	0.00	0.00	14.71	47.06	17.65	5.88	5.88	2.94	5.88	2	>32	21
	T1	0.00	0.00	0.00	0.00	0.00	2.94	38.24	26.47	14.71	2.94	5.88	8.82	4	>32	32
Doxycycline	T0	0.00	2.94	26.47	52.94	8.82	5.88	0.00	2.94	0.00	0.00	0.00	0.00	0.25	0.50	0
	T1	0.00	0.00	5.88	29.41	47.06	5.88	2.94	5.88	0.00	2.94	0.00	0.00	0.50	2	3
Clindamycin	T0	0.00	0.00	2.94	52.94	35.29	5.88	0.00	0.00	0.00	0.00	0.00	2.94	0.25	0.50	3
	T1	0.00	0.00	0.00	23.53	58.82	5.88	0.00	0.00	0.00	0.00	0.00	11.76	0.50	2	12
Augmentin	T0	0.00	0.00	0.00	5.88	14.71	38.24	32.35	2.94	0.00	5.88	0.00	0.00	4	8	N. A
	T1	0.00	0.00	0.00	2.94	8.82	23.53	29.41	11.76	5.88	5.88	8.82	2.94	4	16	N. A
Gentamycin	T0	0.00	0.00	0.00	2.94	17.65	29.41	35.29	2.94	5.88	2.94	0.00	2.94	4	16	6
	T1	0.00	0.00	0.00	0.00	2.94	11.76	41.18	14.71	11.76	5.88	2.94	8.82	4	16	18
Mupirocin	T0	85.29	2.94	8.82	0.00	0.00	0.00	0.00	0.00	0.00	2.94	0.00	0.00	<0.06	0.125	N. A
	T1	67.65	0.00	29.41	2.94	0.00	0.00	0.00	0.00	0.00	0.00	0.00	0.00	<0.06	0.125	N. A
Clarithromycin	T0	0.00	0.00	0.00	0.00	0.00	8.82	58.82	14.71	0.00	5.88	2.94	8.82	2	32	18
	T1	2.94	0.00	0.00	0.00	0.00	14.71	32.35	23.53	5.88	2.94	2.94	14.71	2	>32	26

*T0 and T1 represent the first and second clinical isolate of the longitudinal *S. aureus* pairs. The light-grey fill represents the intermediate susceptible range, and the dark-grey fill represents the resistant range. MIC50 and MIC90 is the minimum inhibitory concentration

(mg/L) that inhibited 50 and 90% of the isolates, respectively. Antibiotics without shading have no interpretive MIC breakpoint accessible.

Figure S1

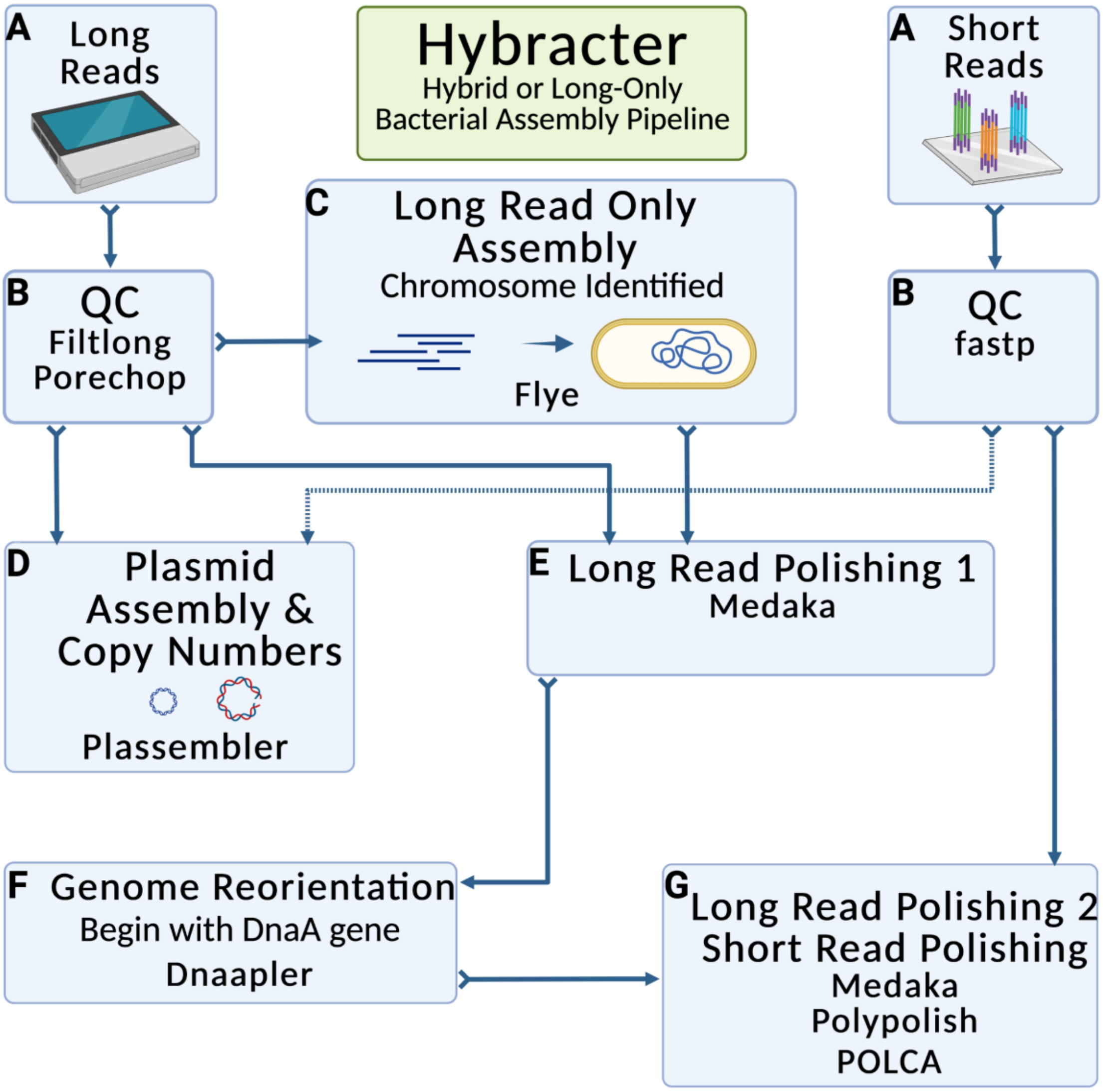

Figure S1 Hybracter workflow. (A) ONT long-reads and paired-end short-reads are specified as input. (B) Long reads are filtered and adapters are trimmed with Filtlong and Porechop. Short reads are filtered and trimmed using fastp. (C) Long-read assembly is conducted using Flye. The bacterial chromosome is identified and extracted for polishing. (D) Plasmids are assembled using a hybrid short-read first method with Plassembler, which also infers the plasmid copy number. (E) The first round of long-read polishing is conducted on the chromosome using Medaka. (F) The chromosome sequence is reoriented, to begin with the dnaA gene with Dnaapler. (G) A second round

of long-read polishing is conducted with Medaka, followed by short-read polishing with Polypolish and POLCA.

Figure S2

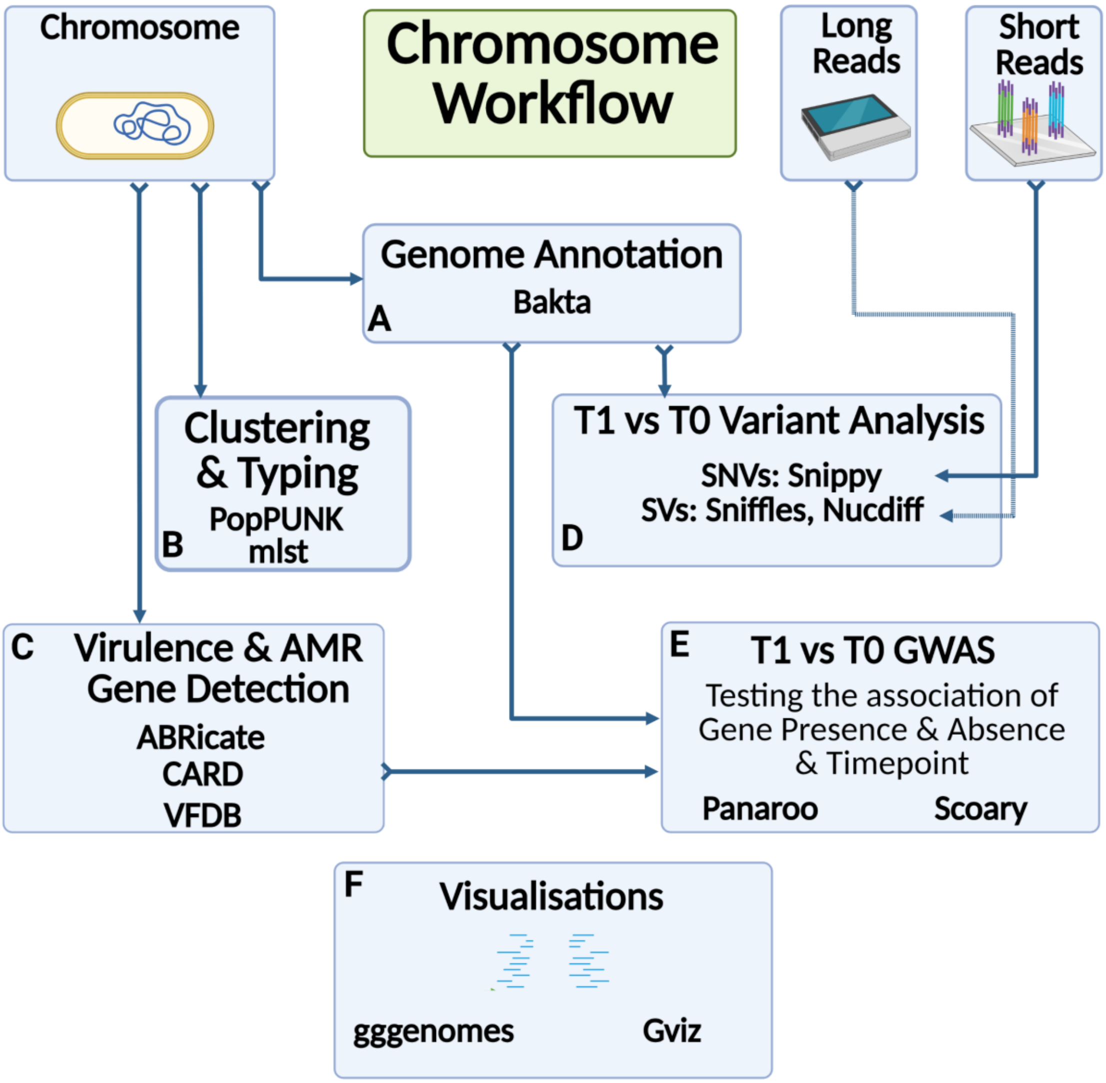

Figure S2 Chromosome analysis workflow. (A) Chromosome genomes annotated with Bakta. (B) Isolates assigned to Variable-length k-mer clusters with PopPUNK and multi-locus sequence types with mlst. (C) Virulence and antimicrobial resistance genes were identified using ABRicate with the VFDB and CARD databases. (D) Small nucleotide identified with short-reads using Snippy. Structural variants were identified using long reads using Sniffles and using the chromosome assemblies using Nucdiff. Gene-wide association study analysis between T0 and T1 isolates was conducted using Panaroo to

create the pangenome and Scoary to test for significance. (F) Visualisations were conducted with gggenomes and Gviz.

Figure S3

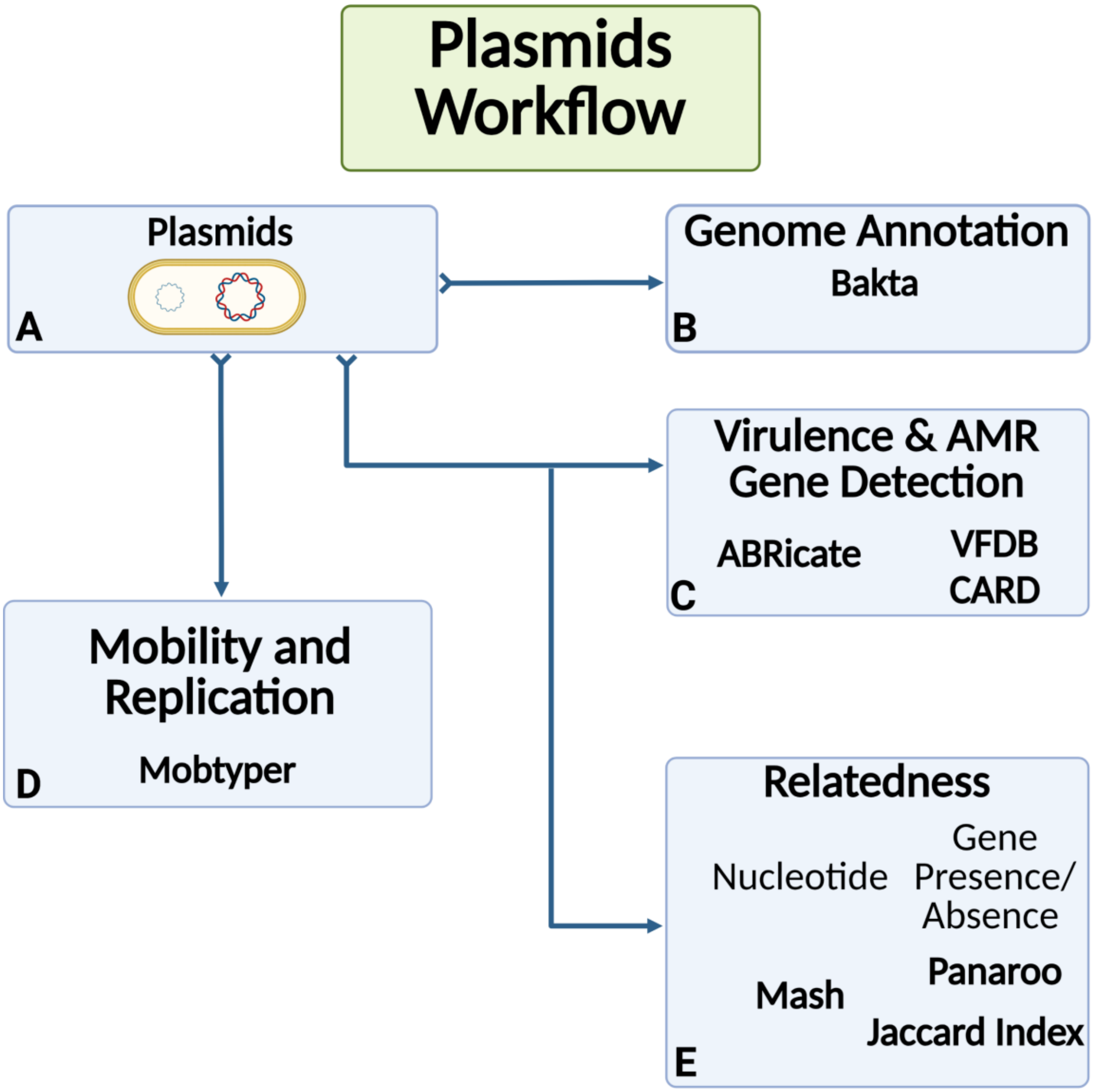

Figure S3 Plasmid analysis workflow. (A) Plasmid assembles from Plassembler as used as input (B) Plasmids are genomically annotated with Bakta. (C) Virulence and antimicrobial resistance genes were identified using ABRicate with the VFDB and CARD databases. (D) Plasmid mobilisation potential is predicted using Mob-Typer to identify potential replicons and relaxases. (E) The relatedness of all plasmids is analysed at the overall nucleotide level by calculating pairwise mash distances between all plasmids, and at the gene presence/absence level by calculating the Jaccard Index of shared genes for each pair of plasmids.

Figure S4

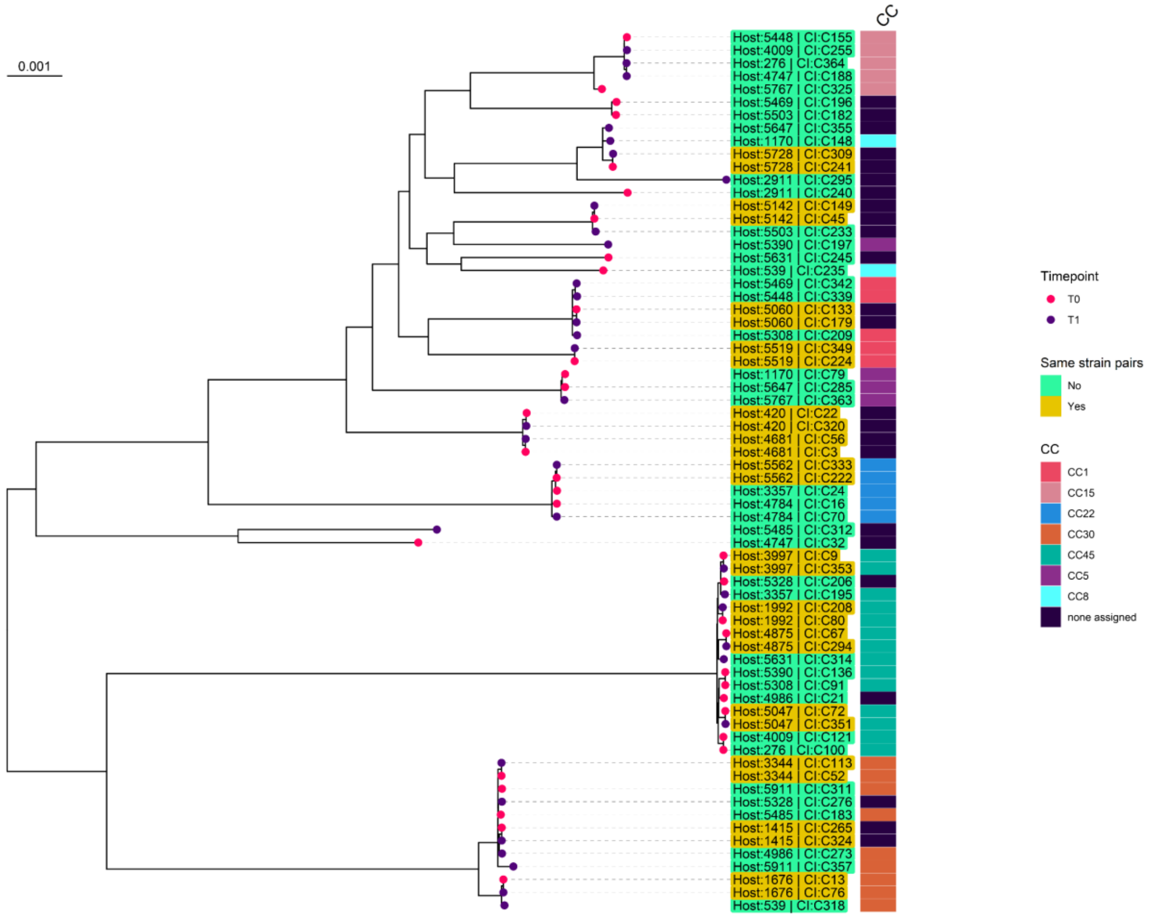

Figure S4 Maximum-likelihood phylogenetic tree depicting the evolutionary relationships among 68 isolates based on core gene alignment. The tree was constructed using the IQ-TREE algorithm with the GTR model and free rate. The X-axis represents nucleotide divergence calculated using uncorrected pair-wise distances. Branch tip colours correspond to the collection timepoints (T0=first, T1=later timepoint), while the right-side colour bar indicates the clonal complex (CC). Branch labels indicate the host ID, and the branch tips are color-coded to indicate the classification of the same or different strain pairs based on the clinical isolate number.

Figure S5

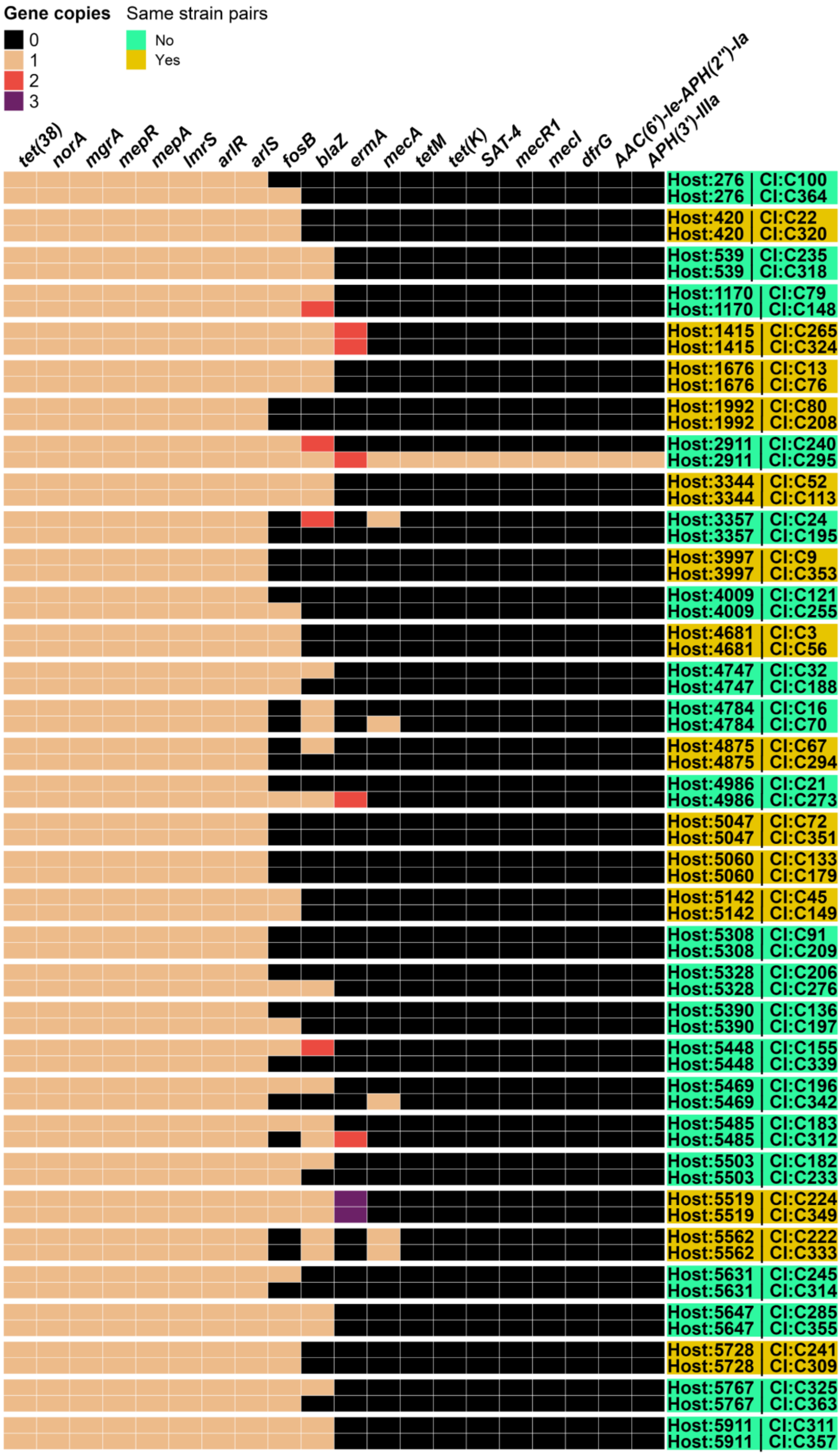
Gene copies
0
1
2
3
Same strain pairs
No
Yes
tet(38)
norA
mgrA
mepR
mepA
lmrS
arlR
arlS
fosB
blaZ
ermA
mecA
tetM
tet(K)
SAT-4
mecR1
mecI
dfrG
AAC(6')-Ie-APH(2'')-Ia
APH(3')-IIIa
Host:276 | CI:C100
Host:276 | CI:C364
Host:420 | CI:C22
Host:420 | CI:C320
Host:539 | CI:C235
Host:539 | CI:C318
Host:1170 | CI:C79
Host:1170 | CI:C148
Host:1415 | CI:C265
Host:1415 | CI:C324
Host:1676 | CI:C13
Host:1676 | CI:C76
Host:1992 | CI:C80
Host:1992 | CI:C208
Host:2911 | CI:C240
Host:2911 | CI:C295
Host:3344 | CI:C52
Host:3344 | CI:C113
Host:3357 | CI:C24
Host:3357 | CI:C195
Host:3997 | CI:C9
Host:3997 | CI:C353
Host:4009 | CI:C121
Host:4009 | CI:C255
Host:4681 | CI:C3
Host:4681 | CI:C56
Host:4747 | CI:C32
Host:4747 | CI:C188
Host:4784 | CI:C16
Host:4784 | CI:C70
Host:4875 | CI:C67
Host:4875 | CI:C294
Host:4986 | CI:C21
Host:4986 | CI:C273
Host:5047 | CI:C72
Host:5047 | CI:C351
Host:5060 | CI:C133
Host:5060 | CI:C179
Host:5142 | CI:C45
Host:5142 | CI:C149
Host:5308 | CI:C91
Host:5308 | CI:C209
Host:5328 | CI:C206
Host:5328 | CI:C276
Host:5390 | CI:C136
Host:5390 | CI:C197
Host:5448 | CI:C155
Host:5448 | CI:C339
Host:5469 | CI:C196
Host:5469 | CI:C342
Host:5485 | CI:C183
Host:5485 | CI:C312
Host:5503 | CI:C182
Host:5503 | CI:C233
Host:5519 | CI:C224
Host:5519 | CI:C349
Host:5562 | CI:C222
Host:5562 | CI:C333
Host:5631 | CI:C245
Host:5631 | CI:C314
Host:5647 | CI:C285
Host:5647 | CI:C355
Host:5728 | CI:C241
Host:5728 | CI:C309
Host:5767 | CI:C325
Host:5767 | CI:C363
Host:5911 | CI:C311
Host:5911 | CI:C357

Figure S5 Gene copy matrix of antimicrobial resistance genes in the genomes of all *S. aureus* clinical isolates (n=68). The isolates are represented by the rows and grouped by pairs and the order of clinical isolate collection. The gene copies of antimicrobial resistance genes are indicated by colour. Labels show the corresponding host ID and the clinical isolate number colour coded with same or different strain pair classification.

Figure S6

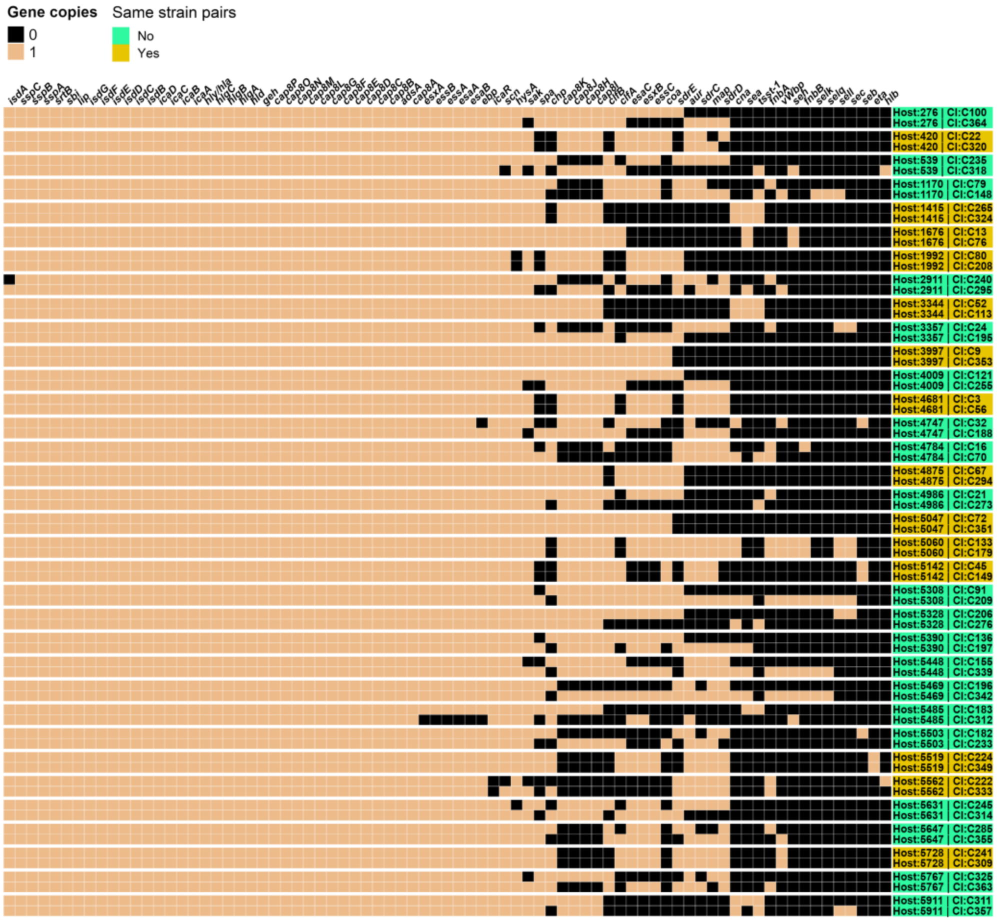

Figure S6 Matrix displaying the virulence genes in the genome of all clinical isolates (n=68). clinical isolates are grouped by pairs and the collection sequence. Labels show the corresponding host ID and the clinical isolate number colour coded with same or different strain pair classification.

Figure S7

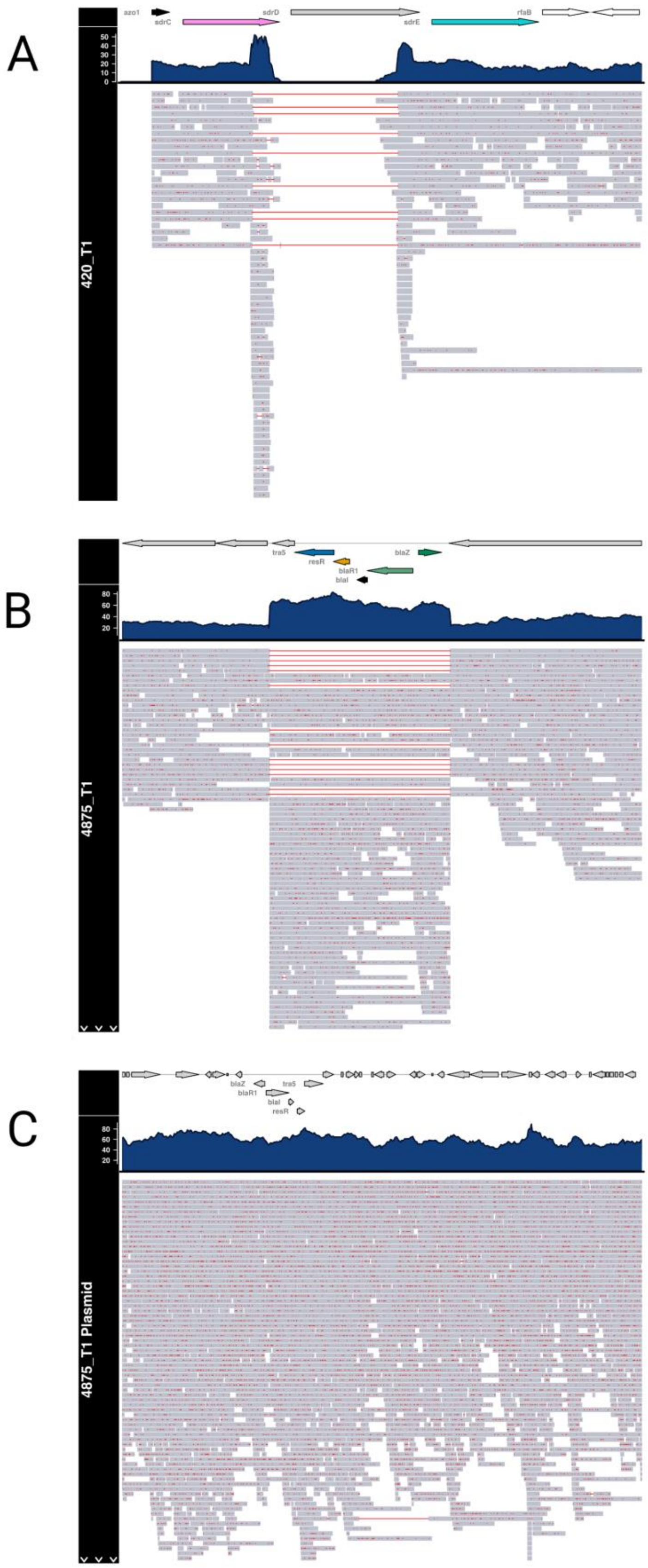
A
azo1
sdrC
sdrD
sdrE
rfaB
50
40
30
20
10
0
420_T1
B
traS
resR
blaR1
blaI
blaZ
80
60
40
20
4875_T1
C
blaZ
blaR1
traS
blaI
resR
80
60
40
20
4875_T1 Plasmid

Figure S7 Coverage and pile-up plot of aligned long-reads (A) The second timepoint isolate of host 420 against the *sdrCDE* locus of the first timepoint isolate, red indicates deleted regions in the reads. (B) The second timepoint isolate of host 4875 against the *β-lactamase* locus of the first timepoint isolate, red indicates deleted regions in the reads. (C) The acquired plasmid of the second timepoint isolate of host 4875, red indicates deleted regions in the reads.

Figure S8

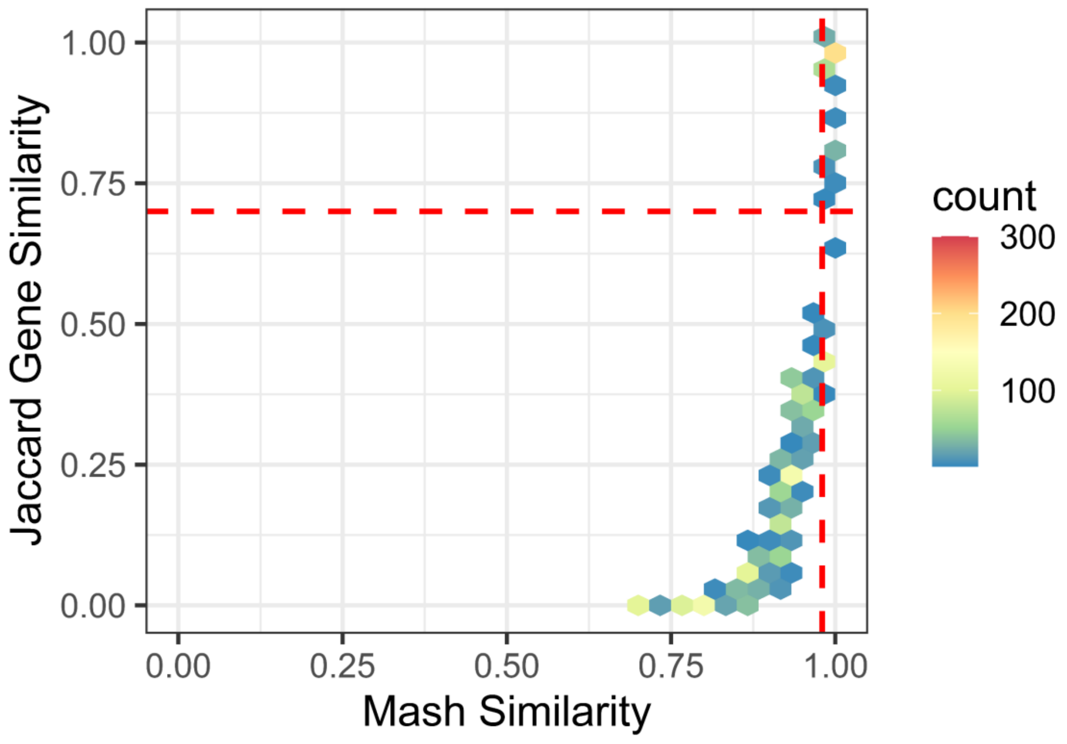

Figure S8 Hexagonal binned plot displaying the relationship between all the plasmids detected based on gene presence and absence and M similarity (n=2,756). The hexagonal cell colour represents the number of data points observed in that cell. Plasmids were considered the same using a threshold of Mash similarity distance greater than 0.98 and Jaccard index of gene presence and absence greater than 0.7.

Figure S9

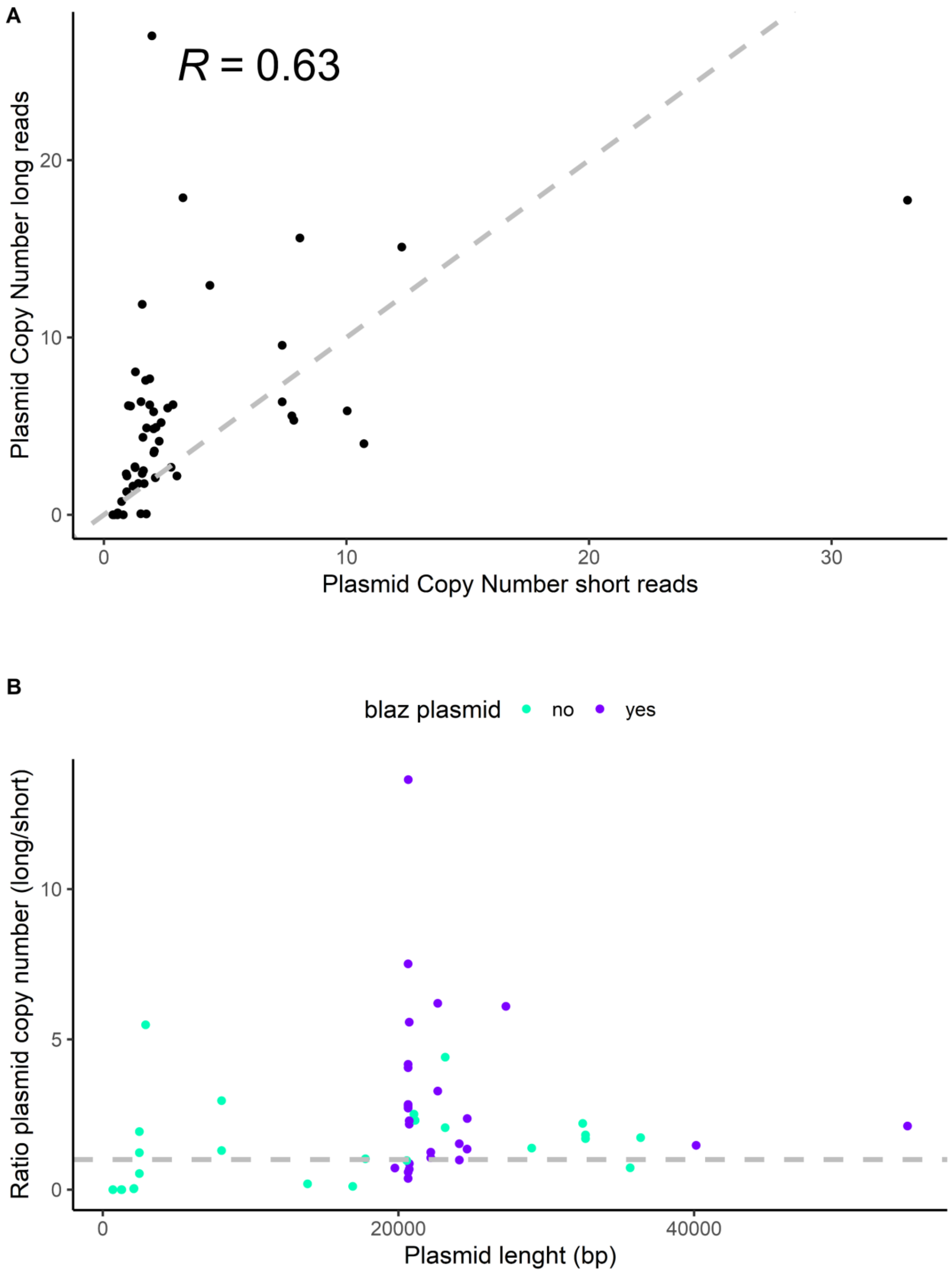

Figure S9 Comparison of plasmid copy numbers estimated from long-read sequencing versus short-read sequencing. (A) Each point represents a single plasmid (n=53), with

the x-axis indicating the copy number from short-read sequencing and the y-axis indicating the copy number from long-read sequencing. The Spearman correlation coefficient is shown in the top left. (B) The ratio of the plasmid copy number (short/long) for all plasmids detected (N=53). *Blaz*-positive plasmids are indicated by colour. The grey line is the intercept of the ratio=1.

Figure S10

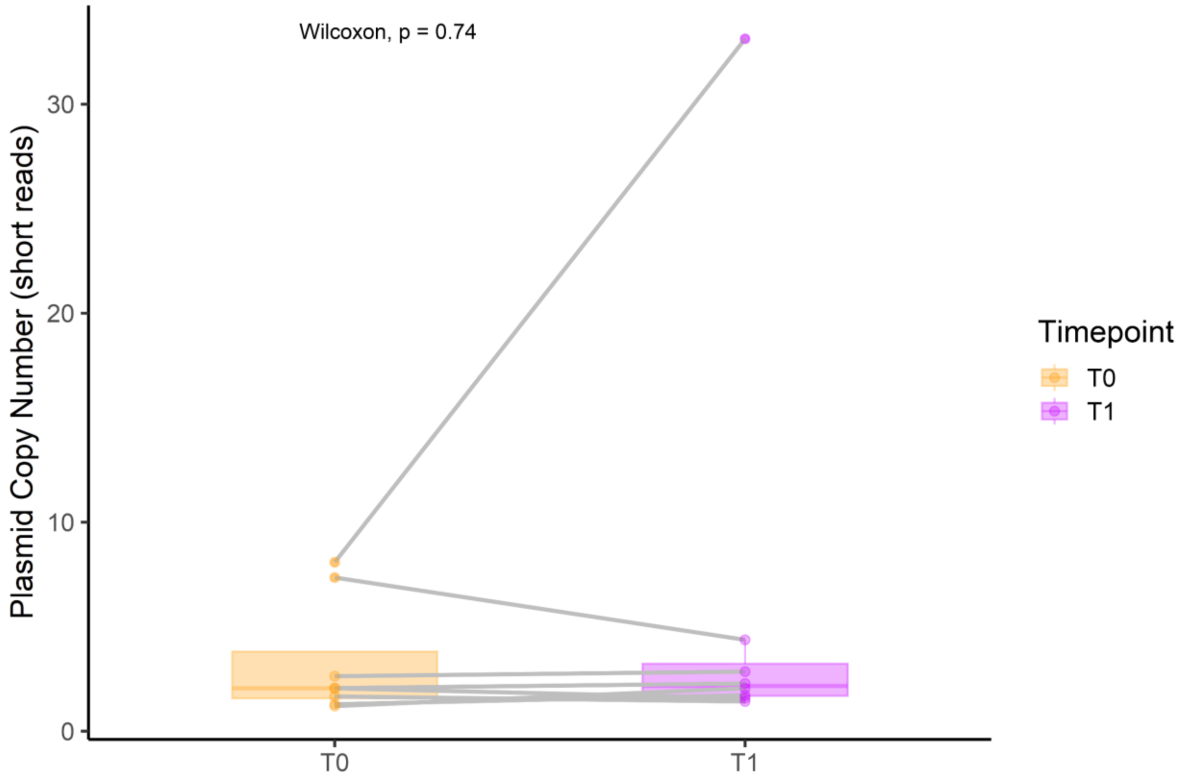

Figure S10 Copy numbers of the conserved plasmids in the 'same strain' group (n=8) for short-read dataset. The colour indicates timepoints, and the grey line indicates paired conserved plasmids. The Wilcoxon signed-rank test compared the copy numbers between the two timepoints, with $p<0.05$ considered significant.

Figure S11

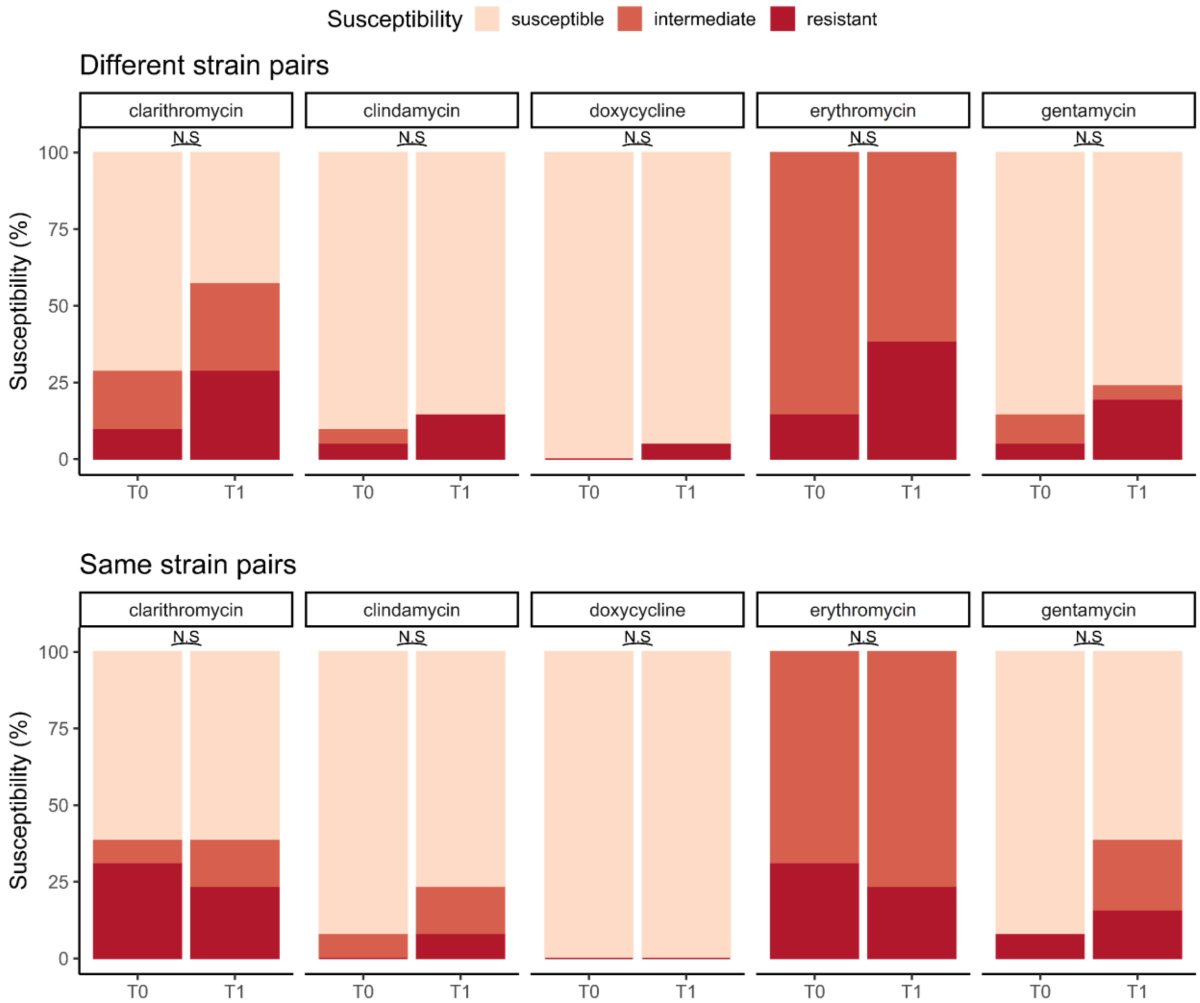

Figure S11 Planktonic antibiotic susceptibility of *S. aureus* isolates (n=68). Antibiotic susceptibility of *S. aureus* isolates is presented based on minimum inhibitory concentration (MIC) breakpoints adapted from the CLSI for isolates classified as the same and different strains (N=14, N=20). Fisher's exact test was used to determine the significant difference in the proportion of resistant and non-resistant isolates between the T0 and T1 groups, with a threshold of $p<0.05$. MIC breakpoints are not available for augmentin and mupirocin.

Figure S12

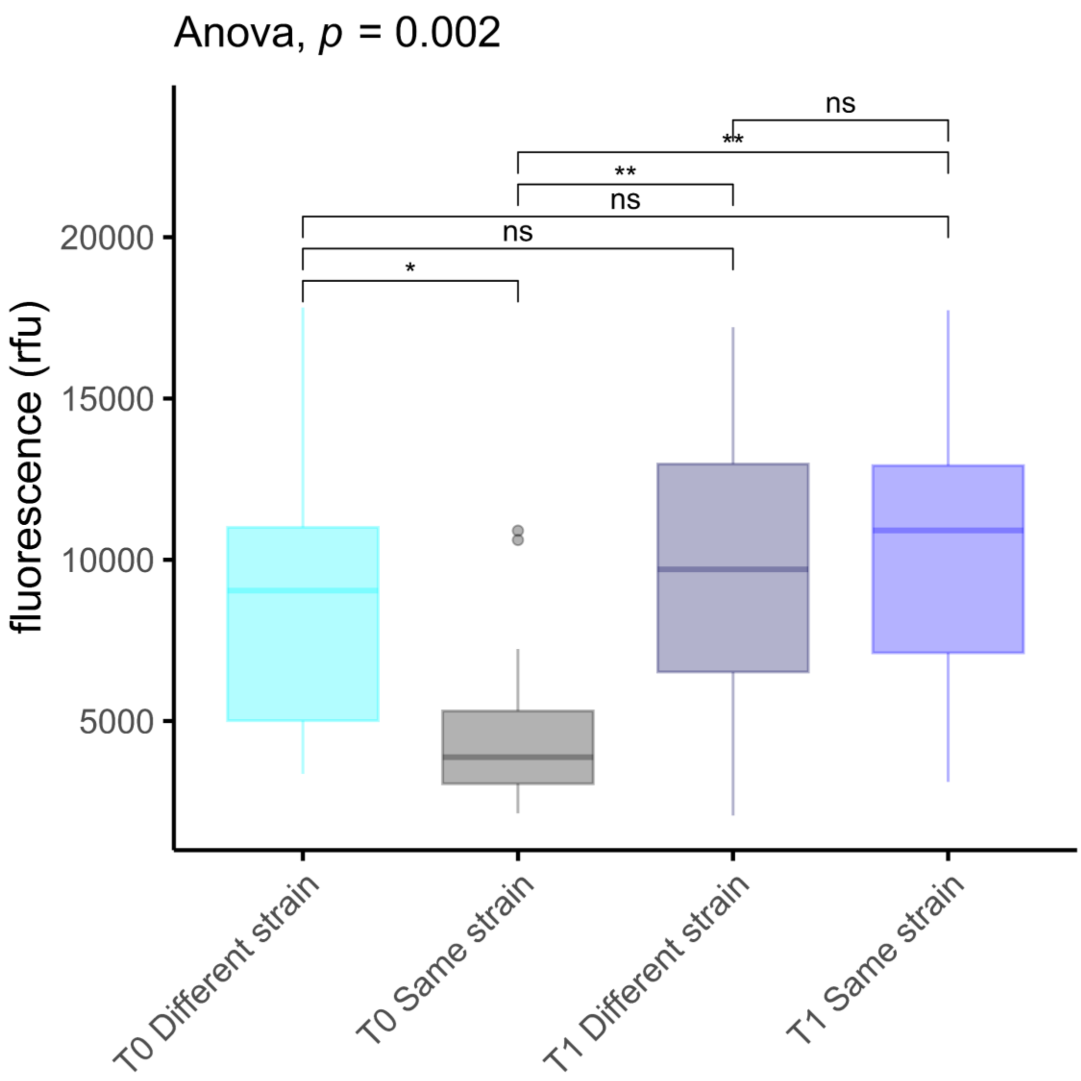

Figure S 12 *S. aureus* biofilms viability after 48 hours of growth for all 68 clinical isolates. The x-axis indicates the first and second clinical isolates classified as the 'same strain' and 'different strain'. Significance was tested using ANOVA, and post-hoc pairwise t-test with Bonferroni correction applied for multiple comparisons.

Figure S13

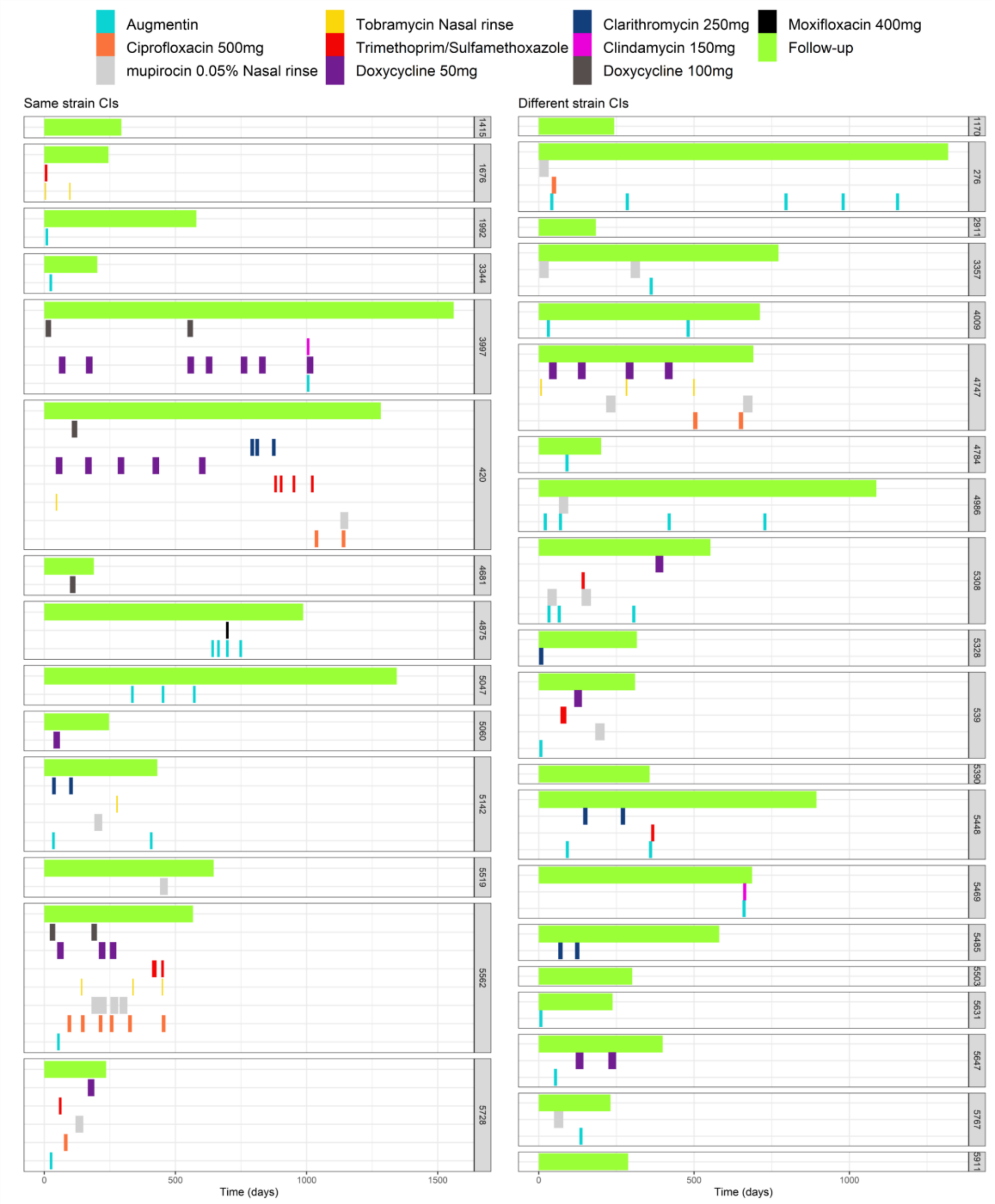

Figure S13 Swimmer plot showing the timeline of antibiotic prescription for each subject between the collection of the first and second timepoint isolates. The antibiotics are indicated by colour. The top green bar represents the time between isolate collection. The left column represents all 'same strain' isolates, and the right column represents 'different strain' isolates. Host ID numbers are indicated on the right side of the columns.

Supplementary text

ST1

Chronic rhinosinusitis (CRS) diagnosis criteria as described by the EPOS:

The presence of two or more symptoms, one of which should be either nasal blockage or nasal discharge with facial pain/pressure or loss of smell. The symptoms should last for more than 12 weeks. Patients were considered difficult-to-treat if no acceptable level of control was achieved despite adequate surgery, intranasal corticosteroid treatment and short courses of antibiotics or systemic corticosteroids in the preceding year of collection.

Asthma status and aspirin sensitivity were collected via self-reported questionnaires at the time of consent for the biobank. Furthermore, an ENT surgeon added the CRS subtype to the biobank after endoscopic assessment.
